# Detection of malignancy in whole slide images of endometrial cancer biopsies using artificial intelligence

**DOI:** 10.1371/journal.pone.0282577

**Published:** 2023-03-08

**Authors:** Christina Fell, Mahnaz Mohammadi, David Morrison, Ognjen Arandjelović, Sheeba Syed, Prakash Konanahalli, Sarah Bell, Gareth Bryson, David J. Harrison, David Harris-Birtill

**Affiliations:** 1 School of Computer Science, University of St Andrews, St Andrews, Scotland, United Kingdom; 2 Pathology Department, NHS Greater Glasgow and Clyde, Glasgow, Scotland, United Kingdom; 3 School of Medicine, University of St Andrews, St Andrews, Scotland, United Kingdom; 4 NHS Lothian Pathology, Division of Laboratory Medicine, Royal Infirmary of Edinburgh, Edinburgh, Scotland, United Kingdom; Universita degli Studi di Milano-Bicocca, ITALY

## Abstract

In this study we use artificial intelligence (AI) to categorise endometrial biopsy whole slide images (WSI) from digital pathology as either “malignant”, “other or benign” or “insufficient”. An endometrial biopsy is a key step in diagnosis of endometrial cancer, biopsies are viewed and diagnosed by pathologists. Pathology is increasingly digitised, with slides viewed as images on screens rather than through the lens of a microscope. The availability of these images is driving automation via the application of AI. A model that classifies slides in the manner proposed would allow prioritisation of these slides for pathologist review and hence reduce time to diagnosis for patients with cancer. Previous studies using AI on endometrial biopsies have examined slightly different tasks, for example using images alongside genomic data to differentiate between cancer subtypes. We took 2909 slides with “malignant” and “other or benign” areas annotated by pathologists. A fully supervised convolutional neural network (CNN) model was trained to calculate the probability of a patch from the slide being “malignant” or “other or benign”. Heatmaps of all the patches on each slide were then produced to show malignant areas. These heatmaps were used to train a slide classification model to give the final slide categorisation as either “malignant”, “other or benign” or “insufficient”. The final model was able to accurately classify 90% of all slides correctly and 97% of slides in the malignant class; this accuracy is good enough to allow prioritisation of pathologists’ workload.

## 1 Introduction

The aim of this work is to automatically sort histopathology whole slide images (WSI) of endometrial biopsies into one of three categories, “malignant”, “other or benign” or “insufficient”. This would allow prioritisation of malignant slides within pathologists’ workload and reduce the time to diagnosis for patients with cancer. Prioritising the small percentage of malignant slides will speed up the diagnosis for patients with cancer, whilst not overly delaying the diagnosis for those without cancer since the benign cases form the majority of the queue.

Cancer of the uterus is one of the top ten most common cancers in women worldwide and the fourth most common in the UK [1]. Endometrial cancers are the most common type of cancer of the uterus [2]. An endometrial biopsy is a key step in diagnosis of endometrial cancer. The tissue obtained from the biopsies is fixed in formalin, then undergoes a series of laboratory processing steps to generate a slide, including staining with Haematoxylin and Eosin (H&E) before being examined by a pathologist and a report generated.

Pathology is increasingly digitised with slides being scanned and viewed on screens rather than through microscopes. The development of artificial intelligence (AI) models in digital pathology, requires data to be available for training and testing the models. The proliferation of WSI has increased the availability of data and led to a boom in the development of AI models for a variety of tasks including automating diagnosis of cancers. The most commonly investigated datasets of H&E WSI are the Camelyon 16 & 17 datasets for breast cancer [3, 4], which have led to hundreds of models with a range of success rates. One of the first AI algorithms with regulatory approval for use on histopathology WSI is for assisting diagnosis of prostate cancer [5].

A recent review of artificial intelligence in gynecological cancers [6] found 13 papers for endometrial cancer, 7 of which used clinical parameters to build models, 3 of which used MRI images, 1 used hysteroscopy images, 1 used slides stained by the Papanicolaou technique from pipelle biopsy [7] and one used H&E slides from endometrial biopsies [8]. In the study using H&E slides of endometrial biopsies, patches of 640 × 480 pixels were extracted from regions of slides that were classified as normal or malignant by pathologists. A convolutional neural network (CNN) was then trained to differentiate patches as endometrial adenocarcinoma and 3 benign classes, normal, endometrial polyp, and endometrial hyperplasia, achieving 93.5% accuracy on the binary classification task and 78.0% sensitivity. The results presented by Sun et al. [8] predict classifications at the patch level rather than at the whole slide level as is undertaken in the current project. Zhao et al. [9] use a global to local CNN to differentiate between types of endometrial hyperplasia, that is changes which can precede the development of cancer, their results are a similar accuracy to pathologists.

A publicly available dataset with endometrial cancer H&E slides on which artificial intelligence papers are published is the Clinical Proteomic Tumor Analysis Consortium (CPTAC) [10] available from the cancer imaging archive, consisting of pathology slides along with genomic data and radiology images. The three studies that used CPTAC aimed to predict the same information as genetic sequencing [11] or illustrate features in H&E slides that could identify different cancer variants [12, 13] and hence allow more personalised treatment. A paper by Song et al. [14] uses a dataset from the cancer genome atlas along with the CPTAC dataset to distinguish between subtypes of endonmetrial cancer. A paper by Zhang et al. [15] aims to split slides into endometrial cancer or not, achieving sensitivity and specificity of 0.924 and 0.801.

The aim of this work is to train a classifier that uses artificial intelligence to automatically classify whole slide images into categories, “malignant”, “other or benign” and “insufficient” at the slide level and to produce heatmaps identifying what parts of the slide are malignant. A classifier of this type could be used to automatically sort slides to prioritise them for pathologists.

## 2 Materials and methods

### 2.1 Ethical approval

This study uses archived samples from the NHS Greater Glasgow and Clyde Biorepository and Pathology Tissue Resource. Patients gave informed consent for surplus tissue to be stored and used for medical research, this consent was recorded in an electronic Surplus Tissue Authorisation form. All data was de-identified within the biorepository and as provided to the researchers was fully anonymized.

- Ethics approval for the study was granted by NHS Greater Glasgow and Clyde Biorepository and Pathology Tissue Resource (REC reference 16/WS/0207) on 4th April 2019.
- Biorepository approval was obtained (application number 511)
- Local approval was obtained from the School of Computer Science Ethics Committee, acting on behalf of the University Teaching and Research Ethics Committee (UTREC) [Approval code- CS15840].

### 2.2 Data

The dataset used in this project includes 2,909 samples from NHS Greater Glasgow and Clyde Biorepository and Pathology Tissue Resource (see Section 2.1 for ethics approval). The categories, subcategories and number of slides in each are shown in Table 1. The samples had examples of five “malignant” subcategories, five “other or benign” subcategories, and a category “insufficient”, where there was insufficient tissue to make a diagnosis. Hyperplasia with atypia was included in the “malignant” category as it is a high risk preinvasive lesion which it is important to detect.

**Table 1:** Number of slides per set, category and subcategory.

Category	Sub-category	Train	Valid	Test	Total
Malignant	Adenocarcinoma	243	113	162	518
	Carcinosarcoma	37	18	28	83
	Sarcoma	11	6	8	25
	Hyperplasia with atypia	106	53	67	226
	Other	4	1	3	8
Malignant	Total	401	191	268	860
Other or benign	Hormonal	158	79	115	352
	Inactive atrophic	170	90	133	393
	Proliferative	184	81	116	381
	Secretory	176	91	116	383
	Menstrual	159	84	111	354
Other or benign	Total	847	425	595	1867
Insufficient	Total	90	44	48	182

The tissue blocks for this study originate from Glasgow Royal Infirmary (NG), Southern General Hospital (SG), Royal Alexandria Hospital (RAH) and Queen Elizabeth University Hospital (QEUH) (all in Glasgow, Scotland) each with independent tissue handling including fixation and tissue processing. New tissue sections were cut from the tissue blocks at one of two different thicknesses (3 microns or 4 microns) and then stained with one of four different H&E protocols. Together, these combinations gave eight different labs maximising WSI variance and thereby decreasing the likelihood of overfitting to any one lab (combination of tissue processing, cutting and staining protocol).

The slides were split into training, validation, and test sets as shown in Table 1. The test set contained the complete groups of slides for two of the labs and these slides were not part of the training and validation sets. The test set then also contained a randomly selected 10% of the slides from the other 6 labs. The remaining 90% of the slides, from the other 6 labs were used for the training and validation sets. Two thirds of these slides were selected randomly for the training set and the rest were used for the validation set. The splits into the test, validation, and training sets were checked to see that there was a balance of the categories and subcategories across the sets. These splits were calculated based on the case labels associated with the samples recorded in the system. During the annotation process these labels were doubled checked and in approximately 5% of the cases the final label associated with the scanned slide was different. This could be because the new slice taken from the sample did not show the same pathology as the original or that the original label was incorrectly recorded. The corrected labels post annotation were the labels that were used for training and testing. This means the final numbers of slides of each type may not match the original percentages described above.

All slides were then scanned at QEUH and saved as WSI. The WSI are hundreds of thousands of pixels in height and width at the highest magnification and are too large to read into memory. Dedicated WSI formats allow access to either small parts of the image at the highest magnification or the whole image at lower magnifications. In this work the slides were scanned using a Phillips Ultra Fast Scanner (UFS) and stored in the isyntax file format. The most detailed view in the WSI is level 0, or 40x magnification where the length of a side of 1 pixel in the image is 0.25*μm*. Higher levels represent lower magnifications in a pyramid where each level is a power of 2 smaller than the previous. For example at level 5, one pixel represents a square patch at level 0 with a length of 2^5^ = 32 pixels per side, or an area of 32 × 32 = 1024 pixels in total.

Variations in tissue handling and staining procedures are common and therefore it was thought worthwhile to try and capture this within the data. It was also decided to hold out two labs in the test set, to test the generalisation to tissue that had been processed differently. This approach does not preclude the use of, nor does it necessarily remove the need for techniques such as stain normalisation or colour augmentations. Further work should test the system against data from different scanners and from different more geographical locations. It is possible that despite this approach to increase the variation in the data there is a wider range used and further techniques to improve generalisation will still be needed.

### 2.3 Annotations

The scanned slides were annotated by a mix of experienced biomedical scientists and pathologists from NHS Greater Glasgow and Clyde. The work of the biomedical scientists was reviewed and approved by a pathologist before use. The annotations took place using the QuPath software [16]; the isyntax [17] files were converted to OMEtiff files using a Glencoe software converter [18] prior to annotation.

**Fig 1:**
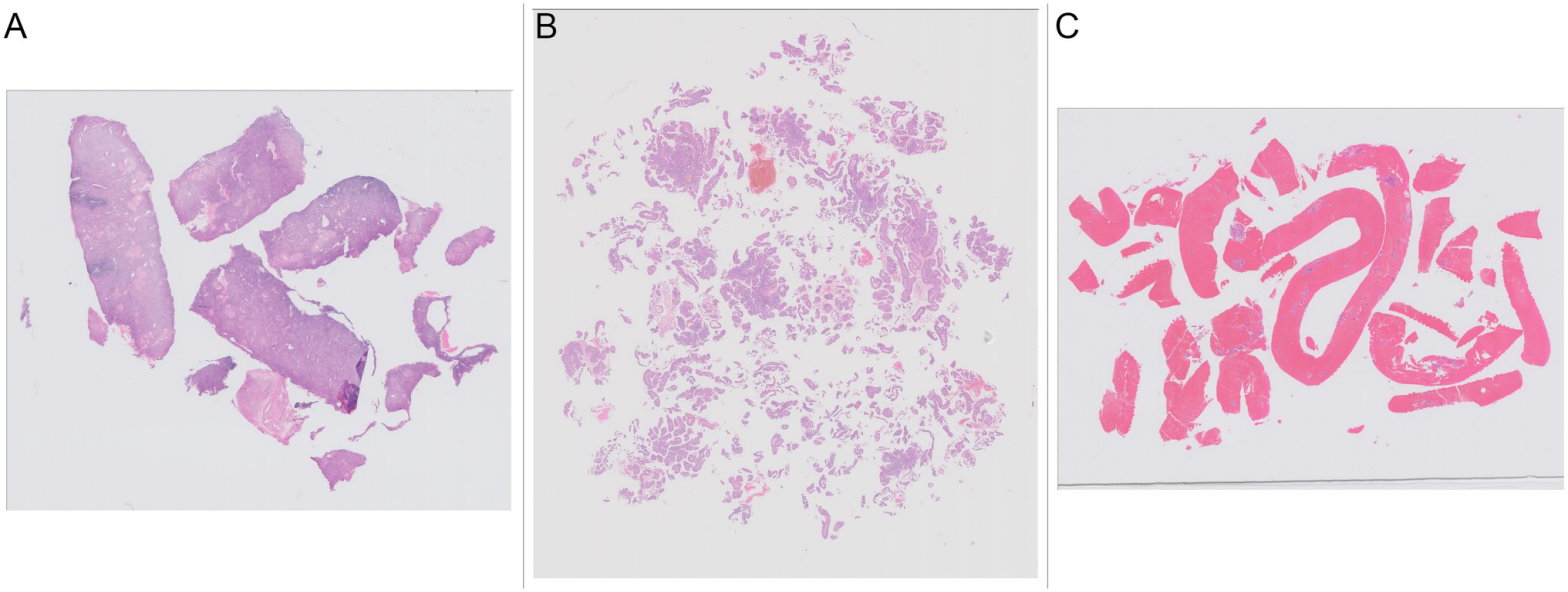
Examples of slides with different amounts and presentation of tissue. (A) Slide with a few large pieces of tissue. (B) Slide with many small fragments of tissue. (C) Slide with large amounts of blood clot.

Many projects when annotating cancerous H&E slides take the approach that everything on the slide is normal apart from annotated areas (This was the approach taken for the widely used Camelyon datasets [3]). Annotation in this study was complicated by the structure of the tissue present on the slides. Some of the slides contained a small number of large contiguous pieces of tissue (Fig 1a), where only annotating the malignant areas is straight forward. However, some of the slides contained a very large number of small fragments of tissue (Fig 1b). These slides would require the pathologists to annotate separately many small bits of tissue on slides where nearly all the tissue was malignant. In addition there was a large amount of blood or mucus that was of no diagnostic value on many slides (Fig 1c). Blood and mucus are not malignant so slides which contain malignant tissue and blood and mucus would require lots of time annotating small bits of tissue within the blood or mucus. Therefore it was decided that annotating blood and mucus either as a separate class or as part of the “other or benign” class would be time prohibitive and an alternate approach was needed.

**Fig 2:**
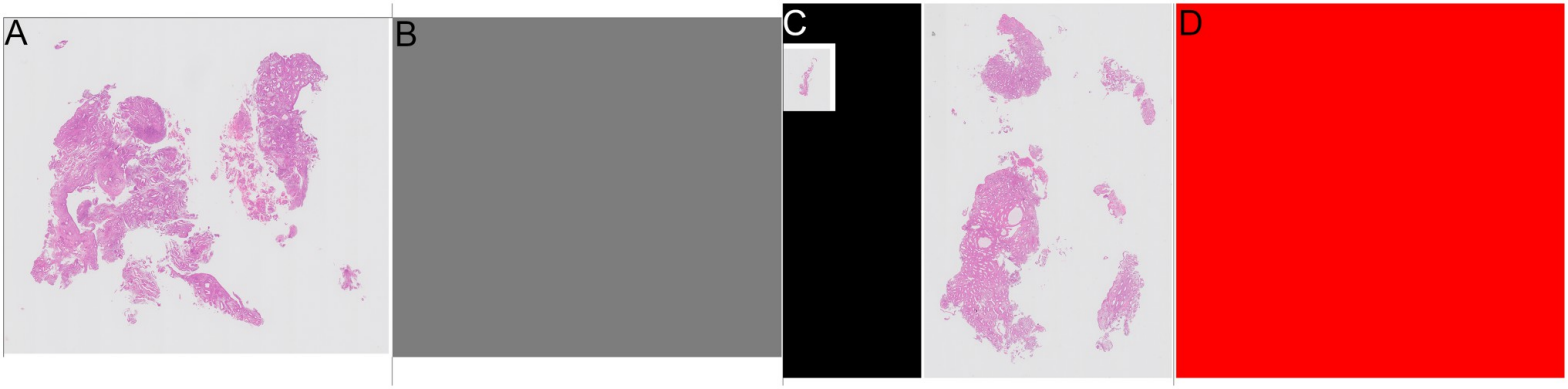
Examples of annotations for slides where all tissue the same category. Grey areas show “other or benign” category, red areas show “malignant” category. (A) Slide where all tissue is “other or benign”. (B) Annotation for slide where all tissue is “other or benign”. (C) Slide where all tissue is “malignant”. (D) Annotation for slide where all tissue is “malignant”.

The annotation approach taken for the current study was to give an overall class to the slide, and then to only annotate parts of the slide that differed from the overall class. The classes used for annotation were “malignant” and “other or benign”. Although there are slides categorised as “insufficient”, these slides are characterised by a lack of tissue rather than a specific type of tissue, so “insufficient” was not used as an annotation class. Annotators were not required to denote the areas of tissue on the slide as tissue detection was applied as part of the preprocessing. Hence, a large number of the annotation files were blank as everything on the slide was from the overall class with no other annotation required. The annotations are visualised in Fig 2 as image masks where grey represents “other or benign” and red represents “malignant”. The overall class provides the background colour and areas with a different class are then coloured appropriately. An example where the whole slide is “other or benign” and the corresponding annotation mask are shown in Fig 2a & 2b. An example where the whole slide is “malignant” and the corresponding annotation are shown in Fig 2c & 2d. Finally an example of a “malignant” slide with some “other or benign” tissue and the corresponding annotation are shown in Fig 4a & 4b.

### 2.4 Algorithm structure

**Fig 3:**
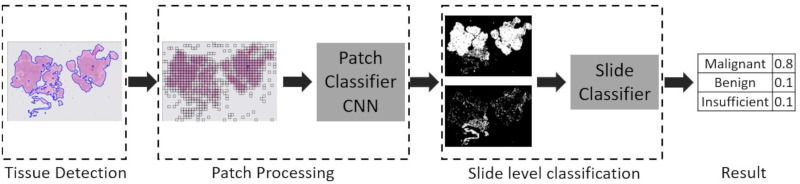
Complete processing pipeline for complete endometrial biopsy WSI.

WSIs are typically in the order of 100,000 pixels per side at the highest magnification. Typical input image sizes of CNNs are in hundreds of pixels, for example the Inception CNN has an input size of 299 × 299 pixels. Therefore the typical approach [19, 20] is to split the WSI into many smaller patches. To further reduce the amount of processing only patches that contain tissue are processed. A CNN is trained to predict the probability of an individual patch being a particular category. The predictions for all the patches from a slide are then reassembled into a heatmap. A slide model is then trained to give an overall prediction for the slide.The complete algorithm (shown in Fig 3) is split into three stages; detection of tissue areas, patch processing, slide level classification, each stage is described in more detail in the following sections.

#### 2.4.1 Detection of tissue areas

**Fig 4:**
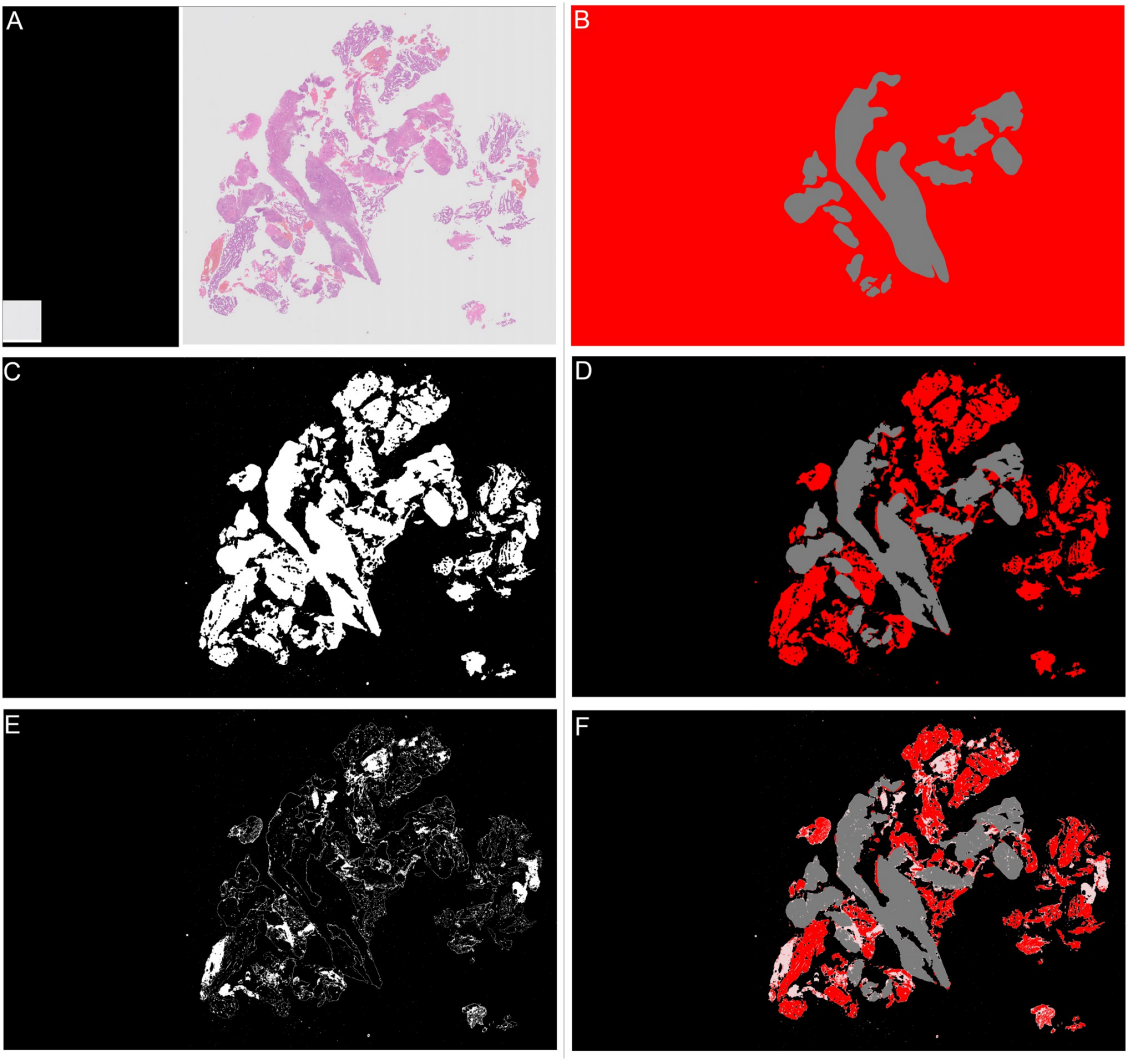
Examples of all stages in slide annotation and detection of tissue. Grey areas show “other or benign” category, red areas show “malignant” category. (A) Thumbnail of “malignant” slide where some tissue is “other or benign”. (B) Annotation for “malignant” slide where some tissue is “other or benign”. (C) Mask showing areas detected as tissue in white, background is shown in black. (D) Combined annotation and tissue detection, black is background, grey is “other or benign”, red is “malignant”. (E) Calculated mask showing areas detected as “blood or mucus” in white, anything that is not “blood or mucus” is shown as black. (F) Combined annotation, tissue and “blood or mucus” detection, black is background, pink is “blood or mucus”, grey is “other or benign”, red is “malignant”.

There are two stages to the detection of tissue areas, background separation and blood and mucus detection. To detect areas of background, firstly a thumbnail image of the slide was taken at level 5. As can be seen in Fig 2c where there are multiple areas of tissue the isyntax format saves these as separate images to reduce filesize. In the thumbnails the missing areas between these images are pure black pixels. Therefore any pixels in the thumbnail that are pure black are converted to pure white. The image was then converted to greyscale and as the background is predominantly white any values of greater than 0.85 were considered to be background. Next a closing transform and a hole filling morphological operation were applied, the operations improve the amount of tissue captured around edges and holes. The mask created by the tissue detection algorithm for the slide shown in Fig 4a is shown in Fig 4c when tissue detection is combined with the annotation (Fig 4b) it gives the areas of the slide as “malignant” or “other or benign” tissue as shown in Fig 4d.

The second stage is to identify any blood or mucus on the slide. Blood and mucus detection is carried out on a pixel by pixel basis. Each of the red, green and blue (RGB) channels are considered separately. A Gaussian filter with a kernel size of 2 is applied. Then a texture filter is applied to each channel both with and without the Gaussian filter to give a total of 12 different features for each pixel (raw pixel value, Gaussian filtered value, texture filter on raw, texture filter on Gaussian filter, for each of 3 channels). A random forest model was trained using a small subset of images with detailed annotations to determine the difference between “blood or mucus” and “tissue” pixels. The trained blood and mucus detection model was then applied to each image to identify “blood or mucus”. For the slide shown in Fig 4a the areas detected as “blood or mucus” are shown in Fig 4e when this is combined with the tissue detection and annotations it gives the areas of the slide as “malignant” or “other or benign” as shown in Fig 4f.

#### 2.4.2 Patch processing

**Fig 5:**
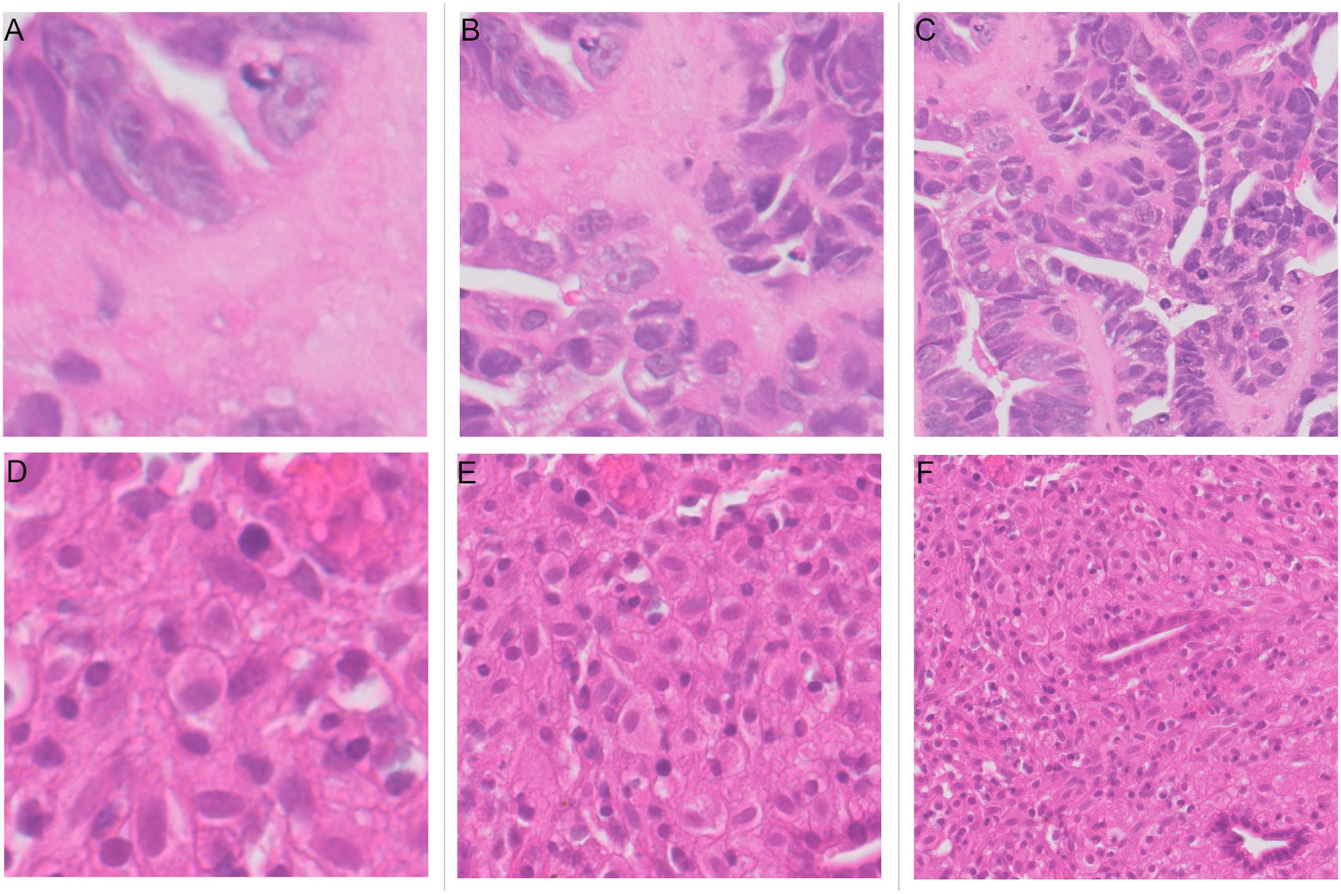
Patches of 256, 512 and 1024 pixels for both “malignant” (upper row) and “other or benign” (lower row) categories. (A) “Malignant” patch of size 128*μm* across (512 × 512 pixels). (B) “Malignant” patch of size 128*μm* across (512 × 512 pixels). (C) “Malignant” patch of size 256*μm* across (1024 × 1024 pixels). (D) “Other or benign” patch of size 64*μm* across (256 × 256 pixels). (E) “Other or benign” patch of size 128*μm* across (512 × 512 pixels). (F) “Other or benign” patch of size 256*μm* across (1024 × 1024 pixels).

Once the tissue detection stage is complete the slide is split into patches on a regular grid. Patches were extracted at the highest resolution (level 0). It was decided to consider different patch sizes because pathologists advised that they would sometimes have difficulty distinguishing between classes when looking at an area of 64*μm* which is equivalent to 256 × 256 pixel patches and therefore larger patches maybe more useful. The size of patches to be extracted was determined by an experiment comparing results at different patch sizes. The stride between patches is set as equal to the size of patches. Three patch sizes were tested 256×256 pixels, 512 × 512 pixels and 1024×1024 pixels. Examples of both “malignant” and “other or benign” patches of the three different sizes are shown in Fig 5 and the results are described in Section 3.1.

A rule is required to decide how much of the patch needs to be tissue for it to be processed. From the detection of tissue step, the mask denoting background, tissue or “blood or mucus” is at level 5 and each pixel in the mask represents 32×32 pixels at level 0. Therefore a 256 × 256 pixel patch at level 0 is 8 × 8 pixels in the mask or 64 pixels in total, each of which can be a different category. Two different rules were investigated to decide if the patch was to be processed. The first was, if there was any tissue in the patch then it was denoted as a tissue patch and was processed. The second was, if tissue was the most common class in the patch then it was denoted as a tissue patch and was processed. The results comparing these two experiments are described in Section 3.2. A python package “wsipipe” [21] has been created to allow the preprocessing code created here to be reused for other projects.

The next step in the pipeline after the patches have been generated is the patch classification CNN. To train the CNN, after the patches have been generated for the they are put into categories of “malignant” or “other or benign”. If the patch contained any “malignant” tissue it was categorised as “malignant” otherwise it was “other or benign”. A balanced training set was created to train a CNN for patch classification. Approximately the same number of patches were found in both the “malignant” and “other or benign” categories. For patches of size 256 × 256 pixels and 512 × 512 pixels 600,000 patches used for training and 300,000 for validation. For patches of 1024 × 1024 pixels there were fewer patches available and so 550,000 patches were selected for training and 260,000 for validation. The CNN used was the GoogLeNet model [22] as implemented in torchvision. A cross entropy loss was used with an SGD optimiser. The initial learning rate was set to 0.01 which was halved every two epochs. Training of the model was stopped when the validation accuracy did not increase for 3 consecutive epochs.

#### 2.4.3 Slide level classification

**Fig 6:**
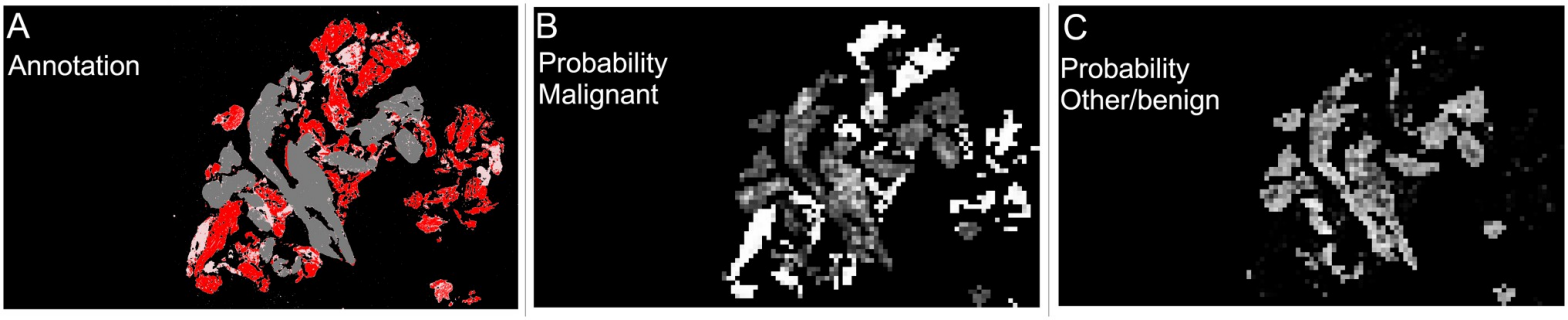
Heatmaps of patch level results for slide shown in Fig 4a, and annotated results for comparison. Slide is from the validation set. (A) Annotation, black is background, pink is “blood or mucus”, grey is “other or benign”, red is “malignant”. This is a repeat of Fig 4f. (B) “Malignant” heatmap of patch results, white is probability of being “malignant” is 1, black is probability of being “malignant” is 0. (C) “Other or benign” heatmap of patch results, white is probability of “other or benign” = 1, black is probability of “other or benign” = 0.

For slide level classification every patch containing tissue on a slide is passed through the trained patch model to predict a probabilities of the patch being in the “malignant”, “other or benign” categories. These predictions are then built into two heatmaps showing the probability of “malignant” and ‘other or benign” categories in the slide based on the position of the patch in the original slide. These heatmaps are greyscale images that are black where the probability of the category is zero and white where the probability of the category is one. For the slide in Fig 4a examples of the heatmaps are shown in Fig 6b (“malignant” category) and Fig 6c (“other or benign” category), they are shown next to the original annotation mask in Fig 6a for easy comparison.

The heatmaps are then used to train a slide level classification model, that predicts a category of “malignant”, “other or benign” or “insufficient”. The results of three different slide classification algorithms are compared in this paper. Two slide classification algorithms based on feature extraction and a third using a CNN based algorithm. Very simple models based on just the numbers of patches and percentage of malignant patches were trialled during the development process but due to the noisy patch classification showed poorer performance than the more complex algorithms outlined below.

#### 2.4.4 Slide classifier developed by Wang et al

The first slide classification algorithm reported, follows the algorithm outlined in the paper by Wang et al. [20], as applied to the Camelyon 16 breast cancer dataset, in which a random forest classifier is used to distinguish between features created from the “malignant” heatmap. Features are created by applying thresholds to the heatmap to create binary masks, which are then segmented. A segment is defined as a connected component, where its pixels are neighbours with another pixel of the same value. Full details of the feature creation are given in Algorithm 1.

The implementation of the Wang algorithm feature extraction in this work, used the scikit image package [23]. The connected components are found using the *measure.label* function with 2-connectivity (pixels neighbours include diagonals). Properties for segments are calculated using the *regionprops* function, the naming in Algorithm 1 follows the definitions in that function. The implementation of random forests used was that in the scikit learn package [24], with 100 estimators as the optimum number, selected via a grid search, all other values remained as the defaults.

**Algorithm 1** Feature creation algorithm based on Wang et al.

1: Find area of tissue in pixels

2: **for** *threshold* = 0.5, 0.6, … 0.9 **do**

3:  Create binary mask of “malignant” heatmap greater than threshold

4:  Calculate “malignant” area as percentage of total tissue area

5:  Calculate total probability of “malignant” pixels in binary mask

6: **end for**

7: Create binary mask of “malignant” heatmap greater than threshold = 0.5

8: Find segments using connected components algorithm.

9: Find the 2 segments with the largest ares

10: **for** *segment* **do**

11:  Calculate area of segment

12:  Calculate eccentricity of segment

13:  Calculate extent of segment

14:  Calculate bounding box area of segment

15:  Calculate major axis length of segment

16:  Calculate maximum probability of segment

17:  Calculate minimum probability of segment

18:  Calculate mean probability of segment

19:  Calculate solidity of segment

20:  Calculate aspect ratio of bounding box of segment

21: **end for**

#### 2.4.5 Slide classifier tuned for endometrial dataset

It was assumed that the Algorithm 1 was tuned for the breast cancer dataset, and so a second algorithm was tuned for this dataset using alternative features, thresholds and numbers of segments. Alternative features were selected from all properties available from the *regionprops* function, where pairs of features were strongly correlated one of each pair was removed. In addition, features that showed no separation between classes were excluded. A similar set of features was also extracted from the heatmap for the “other or benign” class. An XGBoost [25] slide classification model was then trained on these features with parameters of 200 estimators and maximum depth 2 selected via grid search. The full details of this second version are given in Algorithm 2.

**Algorithm 2** Feature creation algorithm tuned for endometrial dataset.

1: Calculate area of tissue in pixels

2: **for** *threshold* = 0.6, 0.9 **do**

3:  Create binary mask of “malignant” heatmap greater than threshold

4:  Calculate “malignant” area as percentage of total tissue area

5:  Create binary mask of “other or benign” heatmap greater than threshold

6:  Calculate “other or benign” as percentage of total tissue area

7: **end for**

8: Create binary mask of “malignant” heatmap greater than threshold = 0.5

9: Find segments using connected components algorithm.

10: Find the 3 segments with the largest area

11: **for** *segment* **do**

12:  Calculate area of segment

13:  Calculate eccentricity of segment

14:  Calculate extent of segment

15:  Calculate bounding box area of segment

16:  Calculate major axis length of segment

17:  Calculate maximum probability of segment

18:  Calculate mean probability of segment

19:  Calculate solidity of segment

20:  Calculate minor axis length of segment

21: **end for**

22: Create binary mask of “other or benign” heatmap greater than threshold = 0.5

23: Find segments using connected components algorithm.

24: Find the 2 segments with largest area

25: **for** *segment* **do**

26:  Calculate area of segment

27:  Calculate bounding box area of segment

28:  Calculate major axis length of segment

29:  Calculate mean probability of segment

30:  Calculate minor axis length of segment

31: **end for**

#### 2.4.6 Slide classifier based on CNN

The heatmaps created from the patch classification are images and therefore it should be possible to use a CNN to classify them into the 3 slide classes. Compared to the many hundreds of thousands of patches available to train the patch classifier there are many fewer heatmaps available to train a slide classifier as there are only 1338 slides in the training set and only 90 of the “insufficient” class. CNNs generally outperform the more traditional machine learning methods such as boosting and random forests when there is enough data, the number of slides available is on the low side, but may still work.

All the heatmaps are different sizes and aspect ratios, therefore some preprocessing is required before they can be used in a CNN. One alternative would be to resize the heatmaps to a standard size, this would distort the heatmaps which may alter features that the CNN would use to distinguish between classes. This approach was rejected because of the “insufficient” slides. The lack of tissue and hence the small size of the heatmaps is clearly a distinguishing trait for these slides. If they were transformed to increase them in size to the same as the other slides, this relative difference in size would be lost. Instead the approach taken was to find the largest heatmap and add zero padding to the other slides so that the size matched the largest slide. The zero padding was added on all sides of the slide so that the original heatmap remained in the centre of the slide. Two heatmaps (“malignant”, “other or benign”) are produced for each slide, each of which is a single channel image, these were stacked together to form a two channel image. Additionally a thumbnail of the slide at the same size was included to form a total 5 channel input to the CNN.

An AlexNet CNN was then trained to use the heatmaps to distinguish between the 3 slide classes. The CNN was trained for 10 epochs using cross entropy loss with an SGD optimiser, a learning rate of 0.0005, which reduced by half after 5 epochs. A random jitter of up to 20 pixels vertically and 40 pixels horizontally was applied to the images. The final model used was from the epoch with the best validation accuracy.

## 3 Results

There are many different options and hyperparameters that can be tuned to optimise performance for classifying endometrial biopsies into “malignant”, “other or benign” and “insufficient”. Here we report the results of the three investigations described in the previous section, patch size, amount of tissue in patches, slide classification algorithm.

There are many ways to measure the performance of algorithms, two values are used, the overall accuracy of slide classification and the percentage of “malignant” slides that are correctly classified. The aim is to use the models created as a tool to prioritise caseloads of pathologists. Therefore it was important to have a tool that can accurately distinguish between types of slides. However, it was considered that it was also important to identify as many of the “malignant” cases as possible. Therefore a fourth investigation was carried out which tuned the final slide classification algorithm to prioritise the percentage of correctly classified “malignant” cases at a cost of incorrectly including some “other or benign” cases and hence lowering overall accuracy.

### 3.1 Patch size selection results

**Fig 7:**
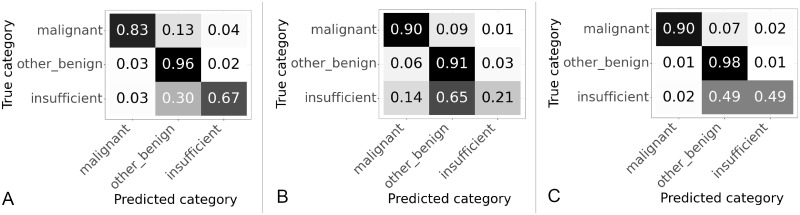
Confusion matrices for results on validation set using different patch sizes. Values are normalized over the true category. (A) Patch size 256 × 256 pixels. (B) Patch size 512 × 512 pixels. (C) Patch size 1024 × 1024 pixels.

Three different patch sizes were tested 256 × 256 pixels, 512 × 512 pixels and 1024 × 1024 pixels, all patches were extracted at level zero. Confusion matrices showing the slide classification results for each patch size are shown in Fig 7, values are normalized over the true category. The overall accuracy and percentage of “malignant” slides correctly categorised for each patch size are given in Table 2.

**Table 2:** Accuracy and percentage of “malignant” slides correctly classified for three different patch sizes.

Patch size	Accuracy	% “malignant” slides correct
256 × 256	90.3	83.2
512 × 512	85.2	89.8
1024 × 1024	92.2	89.8

As the patch size increased from 256 to 512 the percentage of “malignant” slides correctly categorised increased from 83.2% to 89.8%, although the accuracy decreased from 90.3% to 85.2% as shown in Table 2. The accuracy decreased due to problems with classifying “insufficient” slides, this is shown in the confusion matrices in Fig 7a & 7b, where 67% of the “insufficient” slides were accurately classified at 256 × 256 pixels but only 21% of “insufficient” slides were correctly classified at 512 × 512 pixels. For the 1024 pixel patches the sensitivity to “malignant” was the same as 512 and so better than 256, but the accuracy improved from 85.2% to 95.2%. The improvement in accuracy was due to better classification of both “other or benign” and “insufficient” slides as can be seen in Fig 7b & 7c. The classification of “insufficient” slides is still poor probably due to the fact there are many fewer of these slides as shown in Table 1. From these results it appears that the largest patch size of 1024 × 1024 pixels gives highest accuracy and the highest percentage of “malignant” slides categorised correctly.

### 3.2 Results of experiment comparing amount of tissue in patches

**Fig 8:**
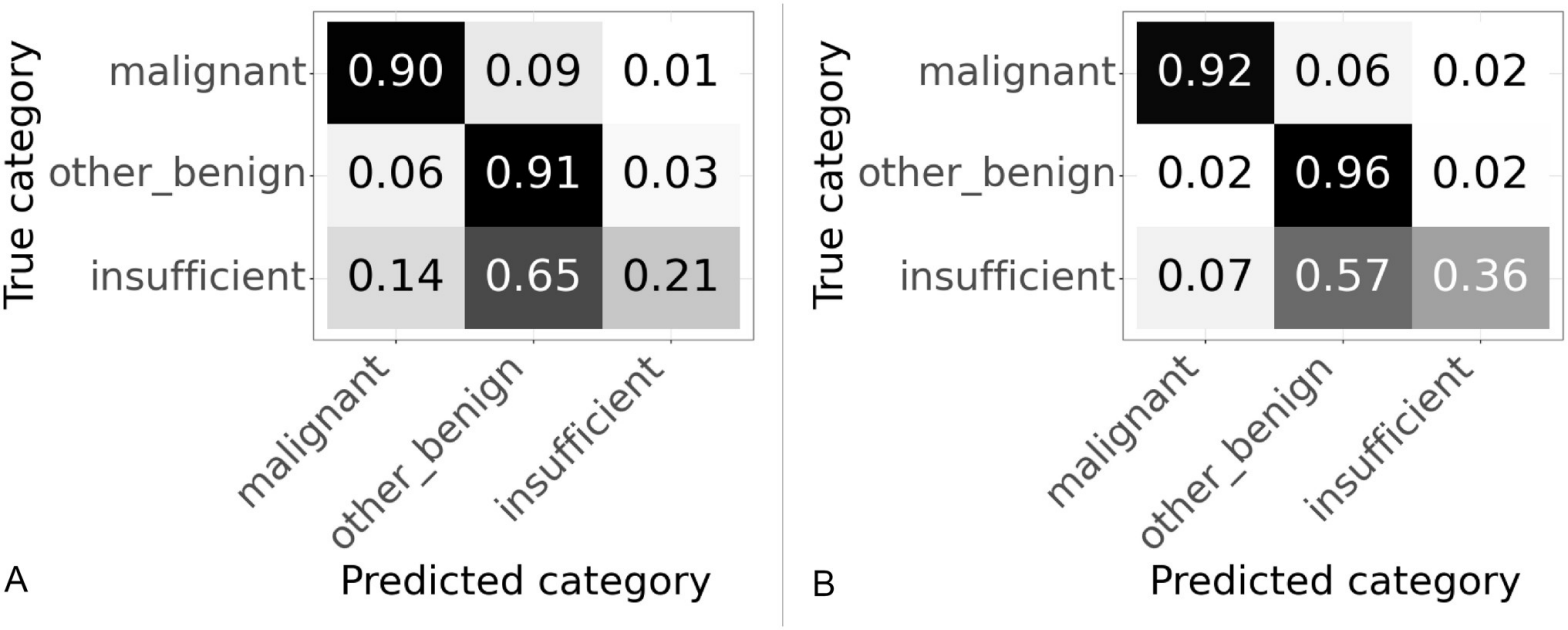
Confusion matrices for patches with different thresholds of tissue used to classify training patches. Results on validation set using 512 × 512. Values are normalized over the true category. (A) Any tissue in patch is a tissue patch. (B) Patch categorised according to largest category present.

Some patches are entirely “blood or mucus”, others are partly “blood or mucus”, partly background, partly tissue. Initially patches in which any part of the patch was classified as tissue were included as tissue patches (that is either “malignant” or “other or benign”). However, it was considered that including patches where the majority of the patch was “blood or mucus” or background with only a small bit of tissue may lead to confusion as there is not enough tissue on the patch for the classifier to work. Therefore it was decide to test changing the algorithm so that training patches were classified as tissue if the majority of the patch was tissue. This was tested using 512 × 512 patches, the normalized confusion matrices for the slide classification results are shown in Fig 8 and the accuracy and percentage of correctly classified “malignant” slides in Table 3.

**Table 3:** Accuracy and percentage of “malignant” slides correctly classified according to threshold of tissue used in training patches.

Threshold for tissue patch	Accuracy	% “malignant” correct
Any tissue	85.2	89.8
Tissue majority in patch	90.8	92.1

Changing to using the majority class improved the accuracy from 85.2% to 90.8% and the percentage of “malignant” slides correctly classified from 89.8% to 92.1% as shown in Table 3. The confusion matrices in Fig 8 show the improvements in accuracy are seen across all classes. From these results it appears that setting the threshold for a patch to be included for classification as the majority class in the patch improves the results.

### 3.3 Slide classifier selection

**Fig 9:**
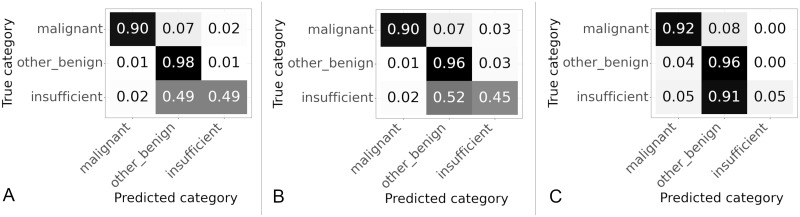
Confusion matrices for results on slide classifier described in Section 2.4.3. Values are normalized over the true category. (A) Slide classifier developed by Wang et al. (B) Slide classifier tuned for endometrial dataset. (C) Slide classifier based on CNN.

The results for different slide classifiers are shown in Fig 9 and Table 4. The three slide classifiers were those described in Section 2.4.3.

**Table 4:** Accuracy and percentage of “malignant” slides correctly classified based on type of slide classifier.

Type of slide classifier	Accuracy	% “malignant” correct
Slide classifier developed by Wang et al.	92.2	89.8
Slide classifier tuned for endometrial dataset	91.1	90.1
Slide classifier based on CNN	89.8	92.7

The best performance in terms of the percentage of the “malignant” class correctly categorised was the CNN based classifier, followed by the endometrial based classifier and then the algorithm based on the breast cancer dataset, for accuracy the order was reversed. For all classifiers the “other or benign” class had the best performance, possibly because this was the largest class with 847 slides available for training compared to less than half that of 401 for the “malignant” class. In comparison the “insufficient” class which had very few slides, 90 in total, available for training. For all the classifiers the performance on the “insufficient” class was poor, the CNN was particular poor only correctly categorising 5% of the “insufficient” slides correctly. Overall the CNN based algorithm was best for the “malignant” class but worst for overall accuracy, with the random forest based algorithm best for overall accuracy but worst for the “malignant” class, with the XGBoost based algorithm balanced in between.

### 3.4 Slide classifier hyperparameter tuning

The slide classifiers were trained to maximise the accuracy. The XGBoost classifier originally had weights of 1 assigned to all classes, thus treating all examples equally and therefore tends to maximise the overall accuracy. In our case the classes are imbalanced with many more “other or benign” examples than “malignant” examples. The “malignant” class has half the number of slides as the “other or benign” class and the “insufficient” class has almost a tenth of the number of slides as the “other or benign” class. Therefore the accuracy is dominated by performance on the “other or benign” class. By weighting the slide classes during training of the slide classifier the performance of the classifier can be tuned to give more weight to the other classes. The weights were tuned using a grid search to find those that correctly classified the highest percentage of “malignant” slides whilst still maintaining a high overall accuracy. Since we care more about getting correct results for the “malignant” samples it therefore makes sense to down weight the other classes. The best performance in the grid search for sensitivity to “malignant” was seen when a weight of 0.05 assigned to the “other or benign” class and a weight of 0.1 assigned to the “insufficient” class.

**Fig 10:**
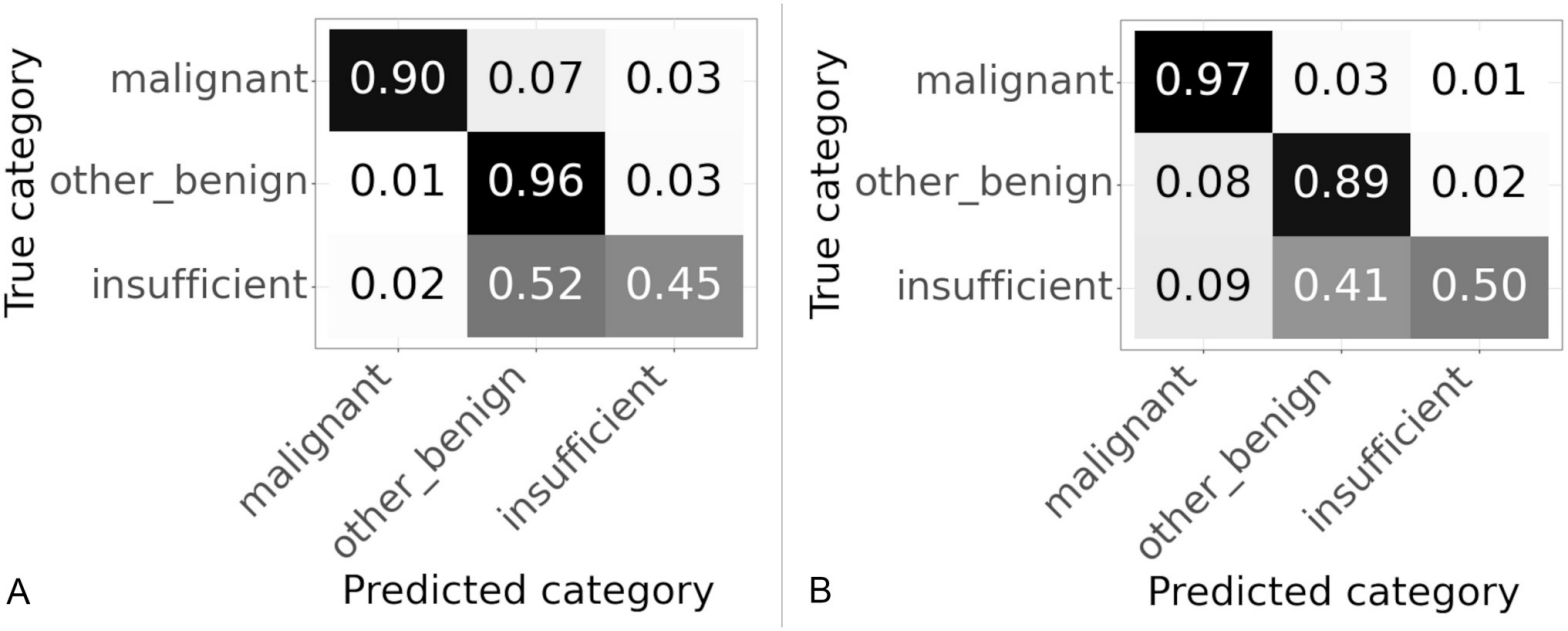
Confusion matrices with slide model hyperparameters tuned to give high percentage of correctly classified “malignant” slides. Results on validation set using 1024 × 1024 patches. Values are normalized over the true category. (A) Original weights, tuned for high accuracy. (B) Weights tuned for high percentage of “malignant” slides correctly categorised.

A set of weights was selected that gave the best performance on the “malignant” class whilst still maintaining a high accuracy. The confusion matrices of the slide classification results for the original weights (effectively those tuned for accuracy) are shown in Fig 10a, are compared to those tuned to correctly predict “malignant” slides in Fig 10b. When comparing to the model trained for best accuracy the model trained for high sensitivity to the “malignant” class had an accuracy of 88.9% compared to the accuracy of 91.1% for the previous model as can be seen in Table 5. However, the percentage of “malignant” slides correctly classified raised from 90.1% to 96.9%. This was the determined to be the best performing algorithm overall.

**Table 5:** Accuracy and percentage of “malignant” slides correctly classified differences based on type of tuning of hyper parameters.

Type of tuning	Accuracy	% “malignant” correct
Best accuracy	91.1	90.1
Best % malignant correct	88.9	96.9

### 3.5 Final model

The final selected model used 1024 × 1024 pixel patches extracted at level 0, with the patch classified as tissue or not according to the majority class within the patch. The slide classifier was used the features of Algorithm 2 with an XGBoost classifier that was tuned for high sensitivity to the “malignant” class. The code for this model has been made available [26].

**Fig 11:**
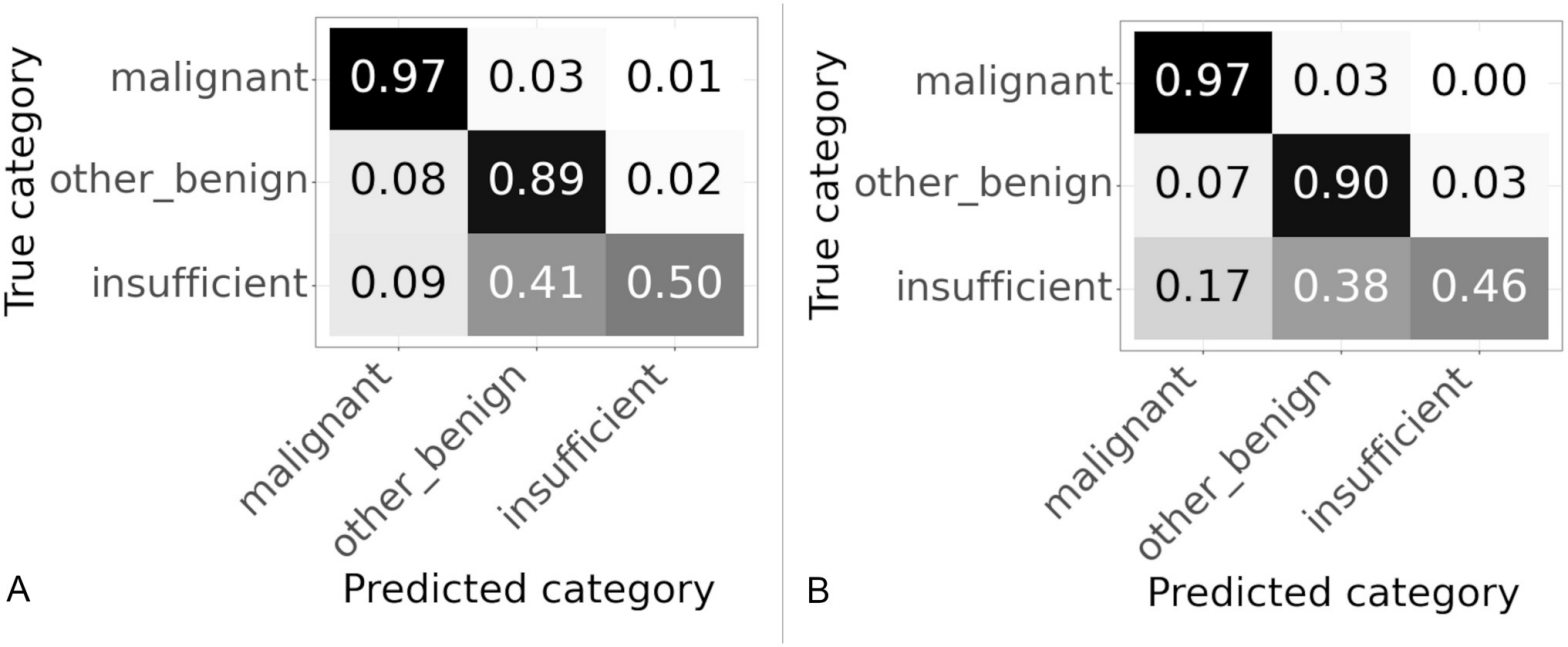
Confusion matrices for slide results on validation and test sets of final model. (A) Results on validation set. (B) Results on test set.

This algorithm was run on both the validation and test sets. The confusion matrices for both datasets are shown in Fig 11. The slides in the test set have never been seen before by the algorithm. The performance of the test set matches closely the performance on the validation set, as can be seen in Table 6. The accuracy on the test set at 89.7% is a slight improvement compared to the 88.9% on the validation set. Whilst the sensitivity to the “malignant” class falls slightly from 96.9% on the validation set to 96.6% on the test set. If the validation and test sets were the same size this reduction in performance equates to one fewer slide is correctly classified on the test set compared to the validation set.

**Table 6:** Accuracy and percentage of “malignant” slides correctly classified for final model on validation and test datasets.

Dataset	Accuracy	% “malignant” correct
Validation set	88.9	96.9
Test set	89.7	96.6

### 3.6 Interobserver variability in annotation

We know that in some cases there may be disagreement even between pathologists as to the correct categorisation of a slide. In order to quantify any disagreement, a subset of 295 of the test slides were annotated separately by the same three pathologists. These observers rated all the slides of the subset, therefore the kappa score was calculated for all pairs of observers and the arithmetic mean of these kappas is reported [27]. It is stated by Hallgren [27] that there is disagreement over interpretation of kappa values, it is however clear that values over 0.8 show an excellent level of agreement. The kappa statistic for each pair of observers was calculated using an unweighted Cohen’s kappa (as implemented in the *cohen_kappa_score* of the scikit learn package [24]). At the category level the kappa statistic, *κ* = 0.95, shows a very high (almost perfect) level of agreement. At the subcategory level the kappa statistic *κ* = 0.89, is also a very high level of agreement.

**Fig 12:**
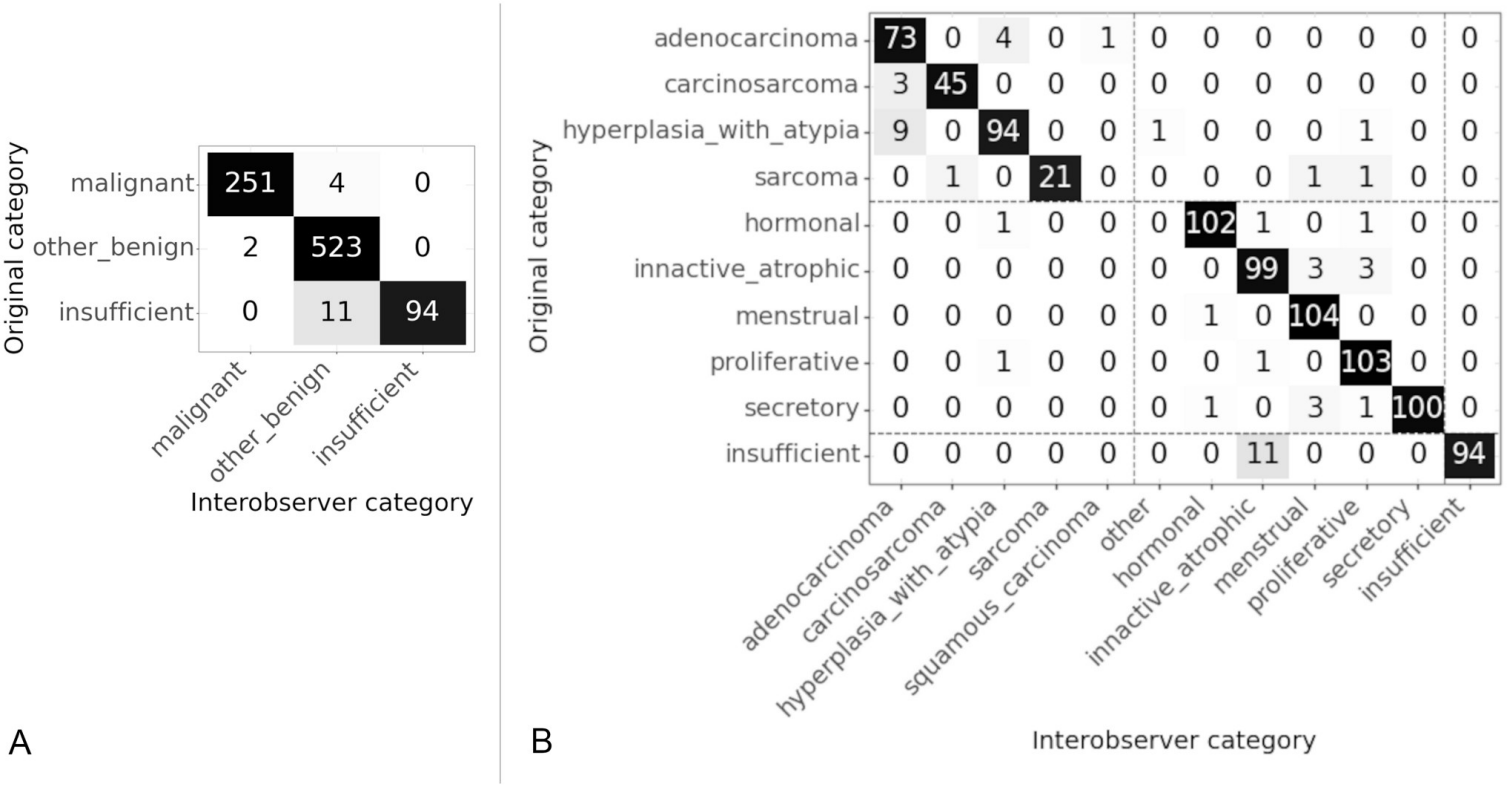
Confusion matrices for interobserver variability. Original category is the label for the slides in the test set. Interobserver category is the label given to the slide by a pathologist in the interobserver test set. (A) Interobserver results at category level, *κ* = 0.95. (B) Interobserver results at sub category level, *κ* = 0.89. Dashed lines show the location of the divide between categories.

The categories and subcategories between which there was disagreement are shown in the confusion matrices in Fig 12. The original category shows the category in which the slides were originally categorised for testing of the AI algorithm. The labels assigned by all observers are concatenated into one list, the counts shown in the matrix are the total numbers and the Interobserver category are the labels of the concatenated list. At the category level we can see that largest number of disagreements is between the “insufficient” and “other benign” class. At the subcategory level results are similar, with most disagreements between “insufficient” and “inactive/atrophic”. However there are nearly as many disagreements between “hyperplasia with atypia” and “adenocarcinoma” both of which are within the “malignant” class.

## 4 Discussion

**Fig 13:**
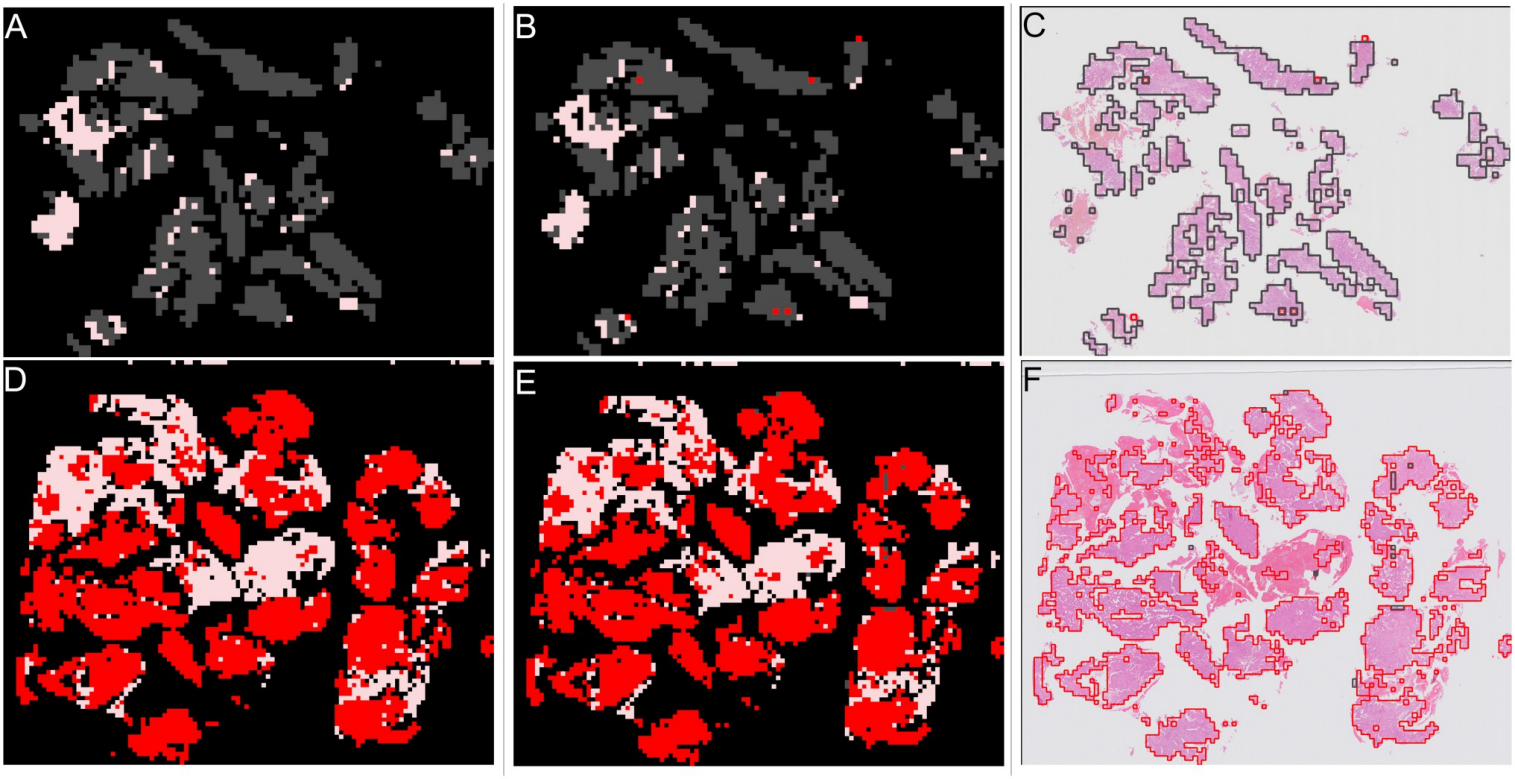
Examples of patch classifications for correctly predicted slides. Top row shows an “other or benign” slide from the validation set, bottom row shows a “malignant” slide from the validation set. First column shows annotation, second column shows model results, third column is model results overlaid on slide image. Red shows “malignant” patches. Grey shows “other or benign” patches. Pink shows “blood or mucus”. (A) Annotation of “other or benign” slide. (B) Patch results of “other or benign” slide. (C) Patch results overlaid on “other or benign” slide image. (D) Annotation of “malignant” slide. (E) Patch results of “malignant” slide. (F) Patch results overlaid on “malignant” slide image.

One advantage of fully supervised models with patch level classification is that they can show the areas that the model directly predicts to be “malignant” or “other or benign”. Commonly used weakly supervised approaches such as multiple instance learning do not directly predict malignant areas, although analogous results such as attention maps can be produced which show parts of the slide that most influence the prediction. Showing clinicians where the model predicts “malignant” can guide them to review the areas of potential concern within the slides, for this reason the fully supervised approach was taken in these experiments. Examples of such output is shown in Fig 13 for an “other or benign” (top row) and a “malignant” slide (bottom row) where the model correctly predicts the slide class. The original annotations are shown in the first column (Fig 13a & 13d), the patch classifications predicted by the model in the second column (Fig 13b & 13e) and finally the classifications overlaid on the thumbnail in the third column (Fig 13c & 13f). In both these cases almost all the patches on the slide are predicted correctly as the same class.

**Fig 14:**
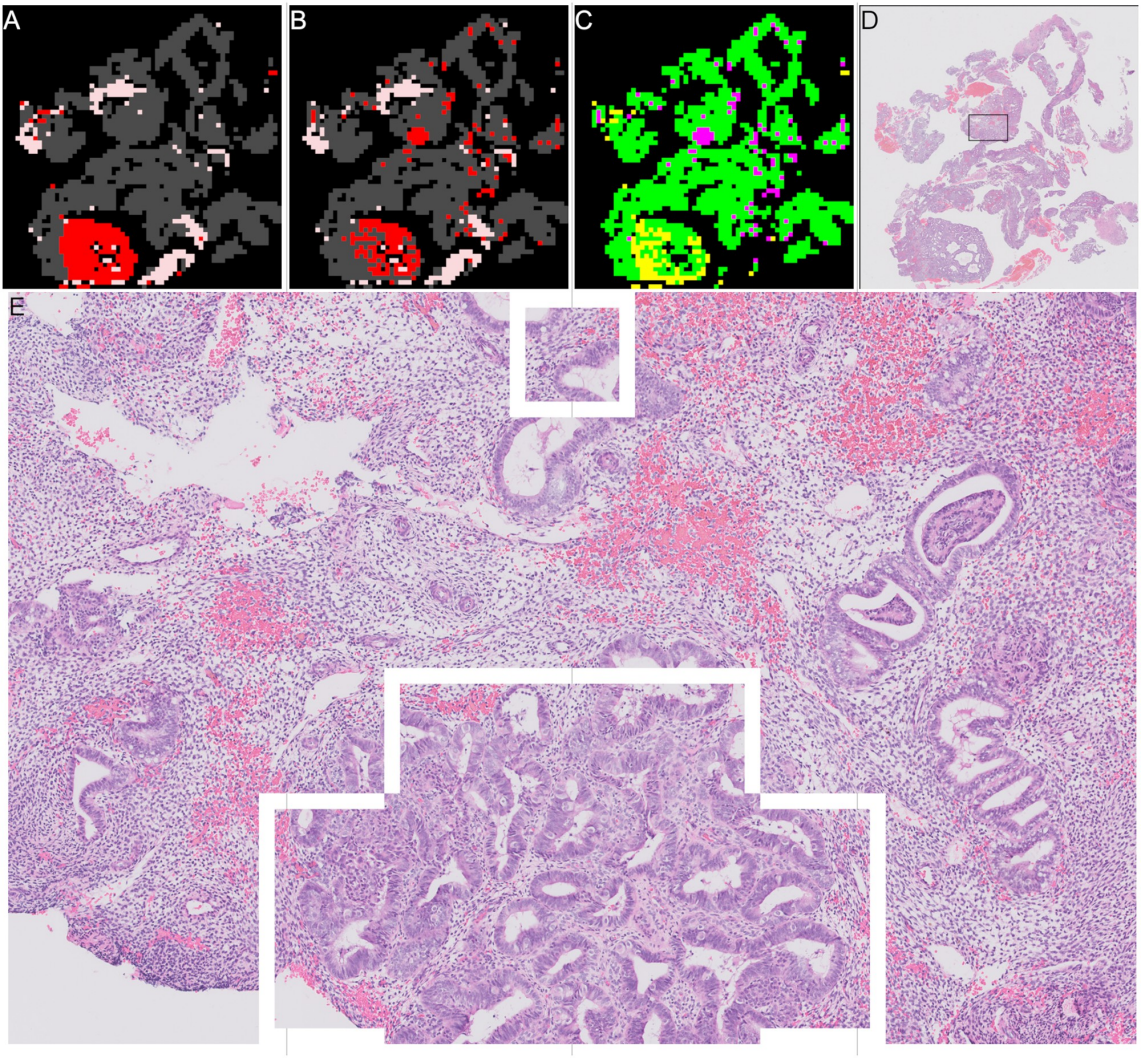
Example of patch classifications for correctly predicted “malignant” slide with some incorrect patches. (A) Annotation of “malignant” slide. Red shows “malignant” patches. Grey shows “other or benign” patches. Pink shows “blood or mucus”. (B) Patch results of “malignant” slide. Red shows “malignant” patches. Grey shows “other or benign” patches. Pink shows “blood or mucus”. (C) Difference between patch results and annotation. Green, prediction correct. Yellow, prediction = “other or benign”, annotation = “malignant”. Magenta, prediction = “malignant”, annotation = “other or benign”. (D) Thumbnail of slide showing region in (E) as black box. (E) Zoomed in detail of region where patch predictions do not match annotations.

A correctly predicted “malignant” slide is shown in Fig 14, compared to the slides in Fig 13 more of the patches are incorrectly classified. Fig 14a shows the annotation from the pathologists with “other or benign” (grey) and “malignant” (red) areas, Fig 14b shows the results of the patch classification. The differences between the annotations and the predictions from the patch classification model are shown in Fig 14c. Correct predictions are coloured green, yellow patches were predicted to be “other or benign” but were annotated as “malignant”, magenta patches were predicted to be “malignant” but were annotated as “other or benign”. We can look at one of the incorrectly predicted areas in more detail, the area shown by the black box in Fig 14d includes an area the model predicts as “malignant” but the annotations show as “other or benign”. This area is shown in more detail in Fig 14e, the white lines outline the area predicted as “malignant”. This slide shows why the slide classification model is needed to convert from the noisy patch predictions and also how the patch classifications could draw clinicians attention to the appropriate areas on slides where there is a mix of tissue types.

Looking at the slides that are incorrectly classified in both the validation and test sets gives an indication of where the algorithm is currently struggling and therefore where it can be improved further. Firstly we discuss a set of slides where issues in the preprocessing pipeline led to the incorrect classification. Secondly, we discuss issues in the patch classification, where the heatmaps generated after patch classification were either wrong or highly unsure. Finally we discuss some future improvements to the slide classifier.

### 4.1 Patch processing issues

Firstly there were a set of slides where issues in the preprocessing pipeline led to the incorrect classification. These issues were in the tissue detection, blood or mucus detection and in the way in which patches were generated. These issues are probably the major contributory factor leading to the incorrect classification of 5 out of the 15 incorrectly classified “malignant” slides.

**Fig 15:**
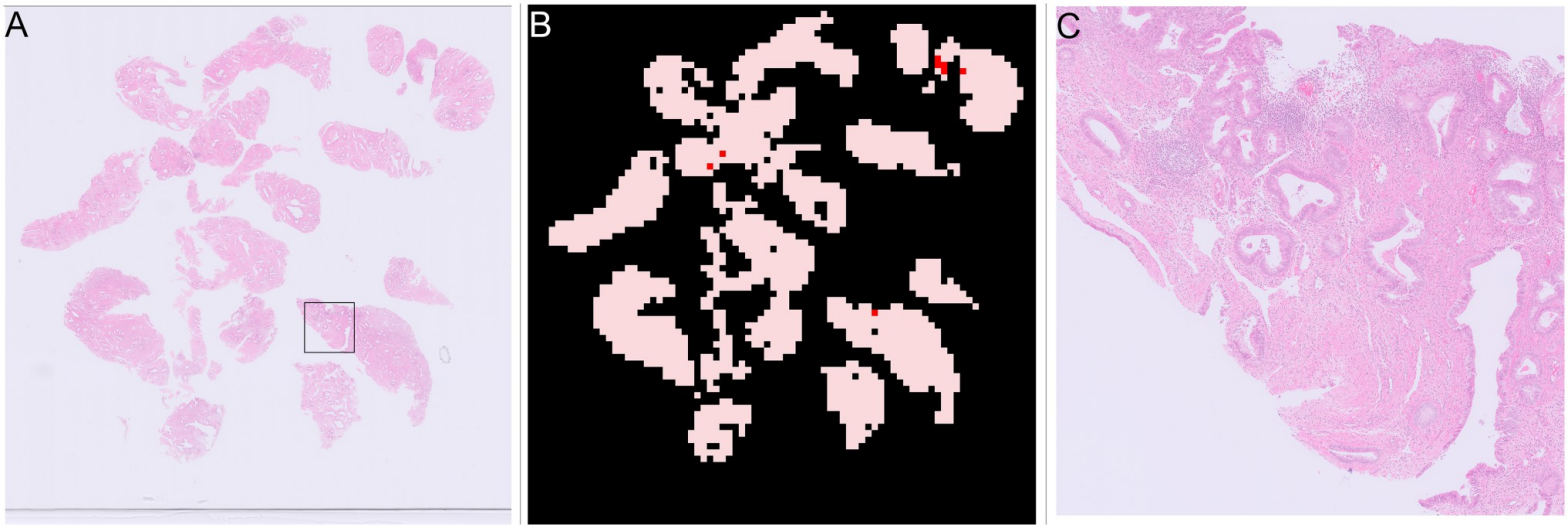
Example of error in blood and mucus detection. (A) Thumbnail of slide with blood and mucus detection problem, square shows location of region in Fig 15c. (B) Mask, areas in pink patches detected as “blood or mucus”, areas in red are tissue. (C) Zoomed in view of area that should be “blood or mucus”, clearly showing tissue.

The blood and mucus detector is over zealous in classing some stained tissue as “blood or mucus”. One clear example of this is shown in Fig 15. The thumbnail of the slide is shown in Fig 15a and the areas detected as “blood or mucus” are shown as pink in Fig 15b, a zoomed in view of one of the areas classified as “blood or mucus” is shown in Fig 15c, which clearly shows tissue rather than “blood or mucus”. As “blood or mucus” patches are not passed into the patch classification process this can lead to tissue being missed and hence disrupting the classification. Improving the blood and mucus classifier would address this problem. This slide is an extreme example, in which the tissue stain is very pale. An alternative that could address this particular case would be to add stain normalisation into the pipeline before blood and mucus detection. Stain normalisation was not investigated further in this set of experiments as there is only one slide with the very pale stain. Stain normalisation could have an added benefit of helping to generalise the model further against staining variations so should be considered as future work.

The blood and mucus detector was trained on a small subset of the training slides that were annotated in detail to include blood and mucus. Examining the slides where it fails and including similar slides in the blood and mucus classification would improve the classifier. However, the detailed annotations required for training the blood and mucus detector are very time intensive, several hours per slide, so are unlikely to be feasible. There are two alternatives to improving the blood and mucus detector, both of which involve passing the “blood or mucus” patches into the patch classifier and so would require extra processing time per slide. One option is to just include all the blood and mucus and allow it as part of the process. As there would be blood and mucus on both “malignant” and “other or benign” slides, the patch classifier should learn that it is not useful to distinguish between the classes and output a probability of approximately 0.5 for both classes. If there is much more blood and mucus present in either the “malignant” or “other or benign” training slides then it might learn “blood or mucus” as a high probability of being one class or another, potentially causing problems. Alternatively the patch classification CNN could be trained to predict 3 classes, “malignant”, “other or benign” and “blood or mucus”, using the currently detected patches as the training set. A 3 category patch classification CNN might then be able to more accurately predict “blood or mucus” for patches that vary from what it has seen before.

**Fig 16:**
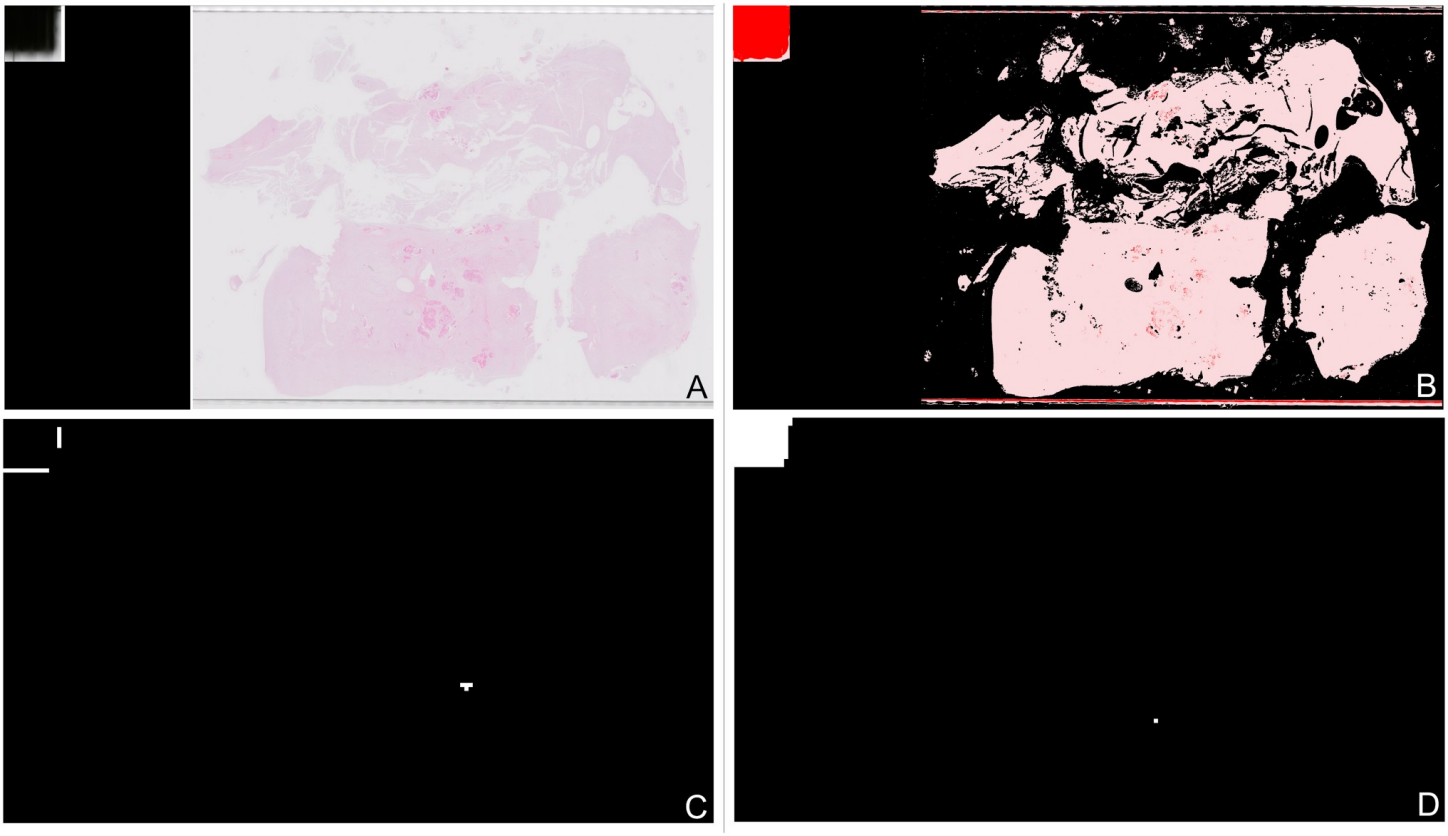
Example of a slide where a tissue detection problem gives an incorrect slide classification, “malignant” slide is classified as “other or benign”. (A) Thumbnail showing slide with small fragments of tissue. (B) Mask showing incorrect area of tissue detection in top left corner of slide. (C) Mask showing “malignant” segments used to extract slide features, two incorrect segments shown in top left. (D) Mask showing “other or benign” segments used to extract slide features, one very large incorrect segment shown in top left.

The tissue detector is not perfect, particularly it struggles with removing bubbles and other inclusions on slides. An example is shown in the thumbnail of a slide Fig 16a where there is a large area in the top left of the slide that is dark background, and the the edge of the slide along the bottom of the image. The red areas in Fig 16b show that these areas are detected as tissue and so are included in the patch classification process. In this slide there are only small amounts of fragmented tissue, therefore these large incorrectly detected areas are more of the heatmaps than the actual tissue. The slide classification process uses the largest segments of each class from the heatmaps, Fig 16c shows two of the largest “malignant” segments are in the top left and Fig 16d shows the largest “other or benign” segment is in the top left. It is therefore unsurprising that the slide classifier incorrectly classifies this slide as “other or benign”.

**Fig 17:**
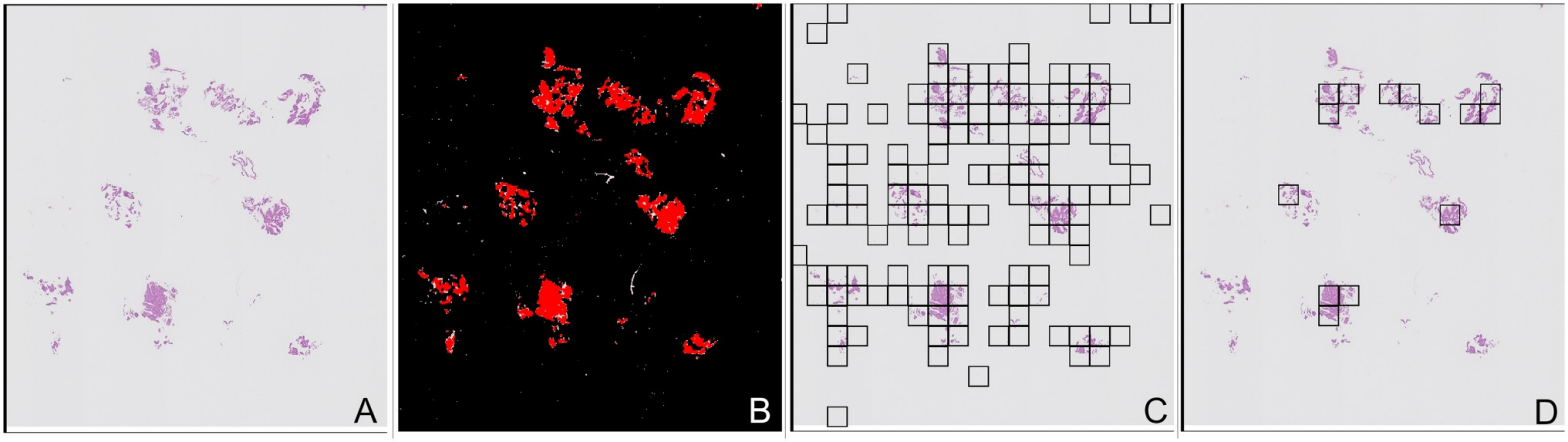
Example of a slide where a patching problem gives an incorrect slide classification. “Malignant” slide is classified as “insufficient”. Black squares shown in Fig 17c, 17d are patches of 1024 × 1024 pixels. (A) Thumbnail showing slide with patching problems. (B) Mask showing tissue detection prior to patching. (C) Tissue patches created when any tissue in a patch creates a patch. (D) Actual tissue patches created as patch has to be more tissue than background, here 10 patches are created.

The third type of issue seen in the preprocessing pipeline was the way in which the patches were generated. The final model used 1024 pixel square patches on a square grid of stride 1024 pixels, with tissue having to be largest classification within the patch (as opposed to background or “blood or mucus”). An example thumbnail of a slide with this problem is shown in Fig 17a, where the small pieces of tissue can be seen. The tissue detection for this slide is shown in Fig 17b showing most of the tissue is correctly detected. When the patches are created on a grid as shown in Fig 17c, because the areas of tissue are small and overlap different patches many of the patches end up getting excluded as most of the patch is background, the remaining patches as shown in Fig 17d. Therefore only a small number of patches are being classified and overall the slide is incorrectly classified as “insufficient” rather than “malignant”. Obviously reducing the patch size and allowing any tissue within the patch to be counted would address this problem, but these criteria were already investigated as discussed in Sections 3.1 and 3.2. An alternative would be to decrease the stride size of the patch grid potentially improving the alignment with the tissue. Alternatively for slides with a small number of patches a minimum number of patches could be randomly positioned within the area detected as tissue.

### 4.2 Patch classification issues

The model is split into two, with a patch classification stage followed by a slide classification stage. Failures at the patch classification stage, have an impact on the slide classification stage. If all patches were correctly classified then there would be no need for the slide classification stage. Whereas if the patches are incorrectly classified then it becomes very difficult for the slide classifier to work. There are 10 “malignant” slides across the validation and test sets where patch classification could be the reason for the incorrect slide classification. These are equally divided between two different problems, where the results of the patch classification is wrong, and where the patch classification results are not showing a clear difference between classes.

**Fig 18:**
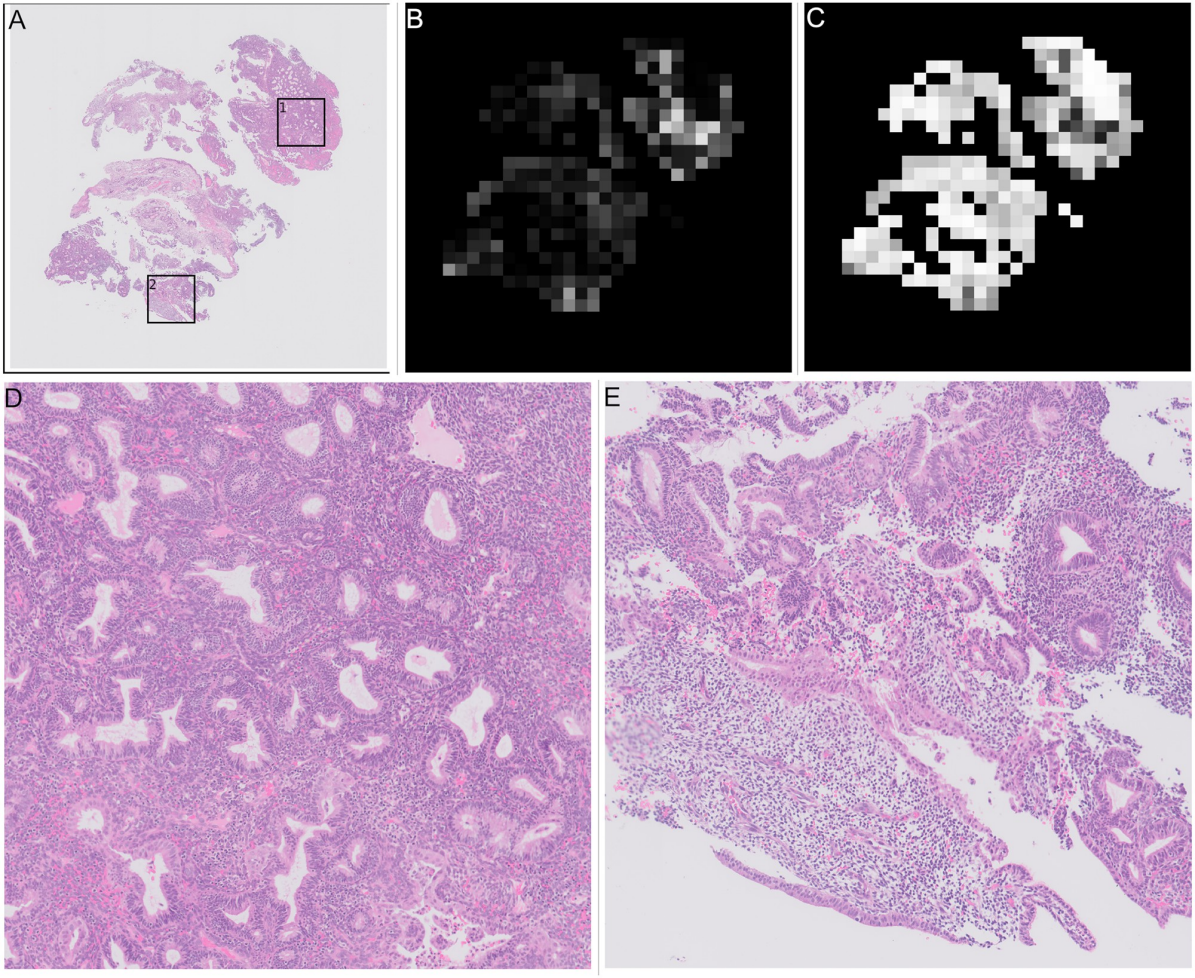
Example of a slide where the output of the patch classifier is wrong. (A) Thumbnail showing “malignant” slide where patch classification is incorrect. Square 1 shows area in Fig 18d. Square 2 shows area in Fig 18e. (B) Results of “malignant” heatmap patch classification showing probability of “malignant”, white = 1, black = 0. (C) Results of “other or benign” heatmap patch classification showing probability of “other or benign”, white = 1, black = 0. (D) Zoomed in on area with highest probability of being “malignant” (square 1). (E) Zoomed in on area with highest probability of being “other or benign” (square 2).

An example of a slide where the patch classification is wrong is seen in Fig 18, this is a “malignant” slide and all the tissue shown in the thumbnail of Fig 18a was annotated as “malignant”. As can be seen in Fig 18c, the patch classifier classified most of the tissue as “other or benign” shown by the white areas with a high probability of being “other or benign” as opposed to the probabilities of being “malignant” shown in Fig 18b where very few white areas are shown. The areas in the squares shown in Fig 18a are shown in more detail in Fig 18d & 18e, square number one is the area with highest probability of being “malignant” and square number two is an area with high probability of being “other or benign”. In the more detailed images it can be seen that although the patches are classed as “other or benign” the tissue is “malignant”. This is likely to reflect a limitation in the annotations, annotations from humans whilst excellent are a gold standard and are not a perfect ground truth. It could be within other slides there are areas that have been missed by annotators. This would not necessarily affect the overall slide classification, but would complicate measurements of patch classification accuracy.

**Fig 19:**
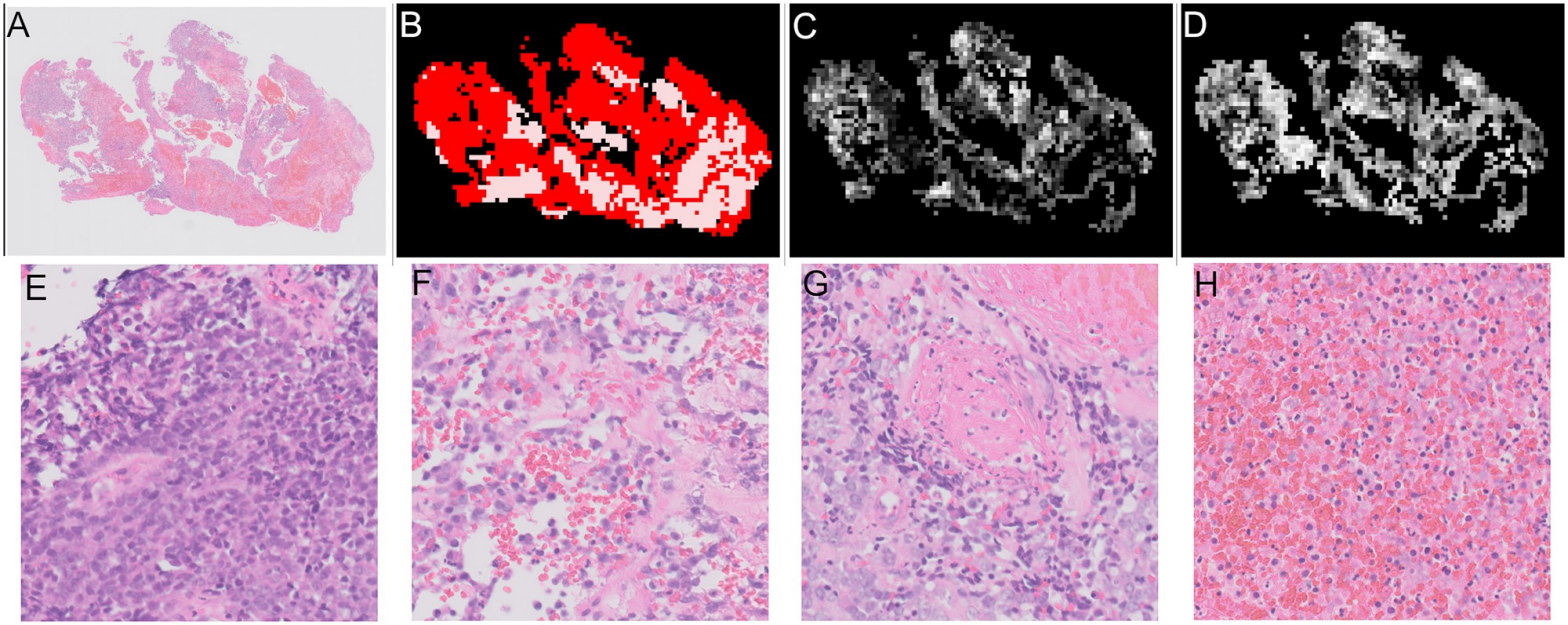
Example of a slide where patch classification is unsure. (A) Thumbnail showing slide where patch classification is unsure, “malignant” slide. (B) Mask showing areas of “malignant” tissue in red and “blood or mucus” in pink. (C) Results of patch classification showing probability of “malignant”, white = 1, black = 0. (D) Results of patch classification showing probability of “other or benign”, white = 1, black = 0. (E) Example “malignant” patch probability “malignant” = 0.980 (F) Example “malignant” patch probability “malignant” = 0.502 (G) Example “other or benign” patch probability “malignant” = 0.498 (H) Example “other or benign” patch probability “malignant” = 0.008.

An example of a slide where the patch classification are not showing clear differences between categories is shown in Fig 19. The thumbnail is shown in Fig 19a and mask showing “malignant” tissue areas in Fig 19b. As can be seen in the results for probability of patches being “malignant” in Fig 19c and the probability of a patch being “other or benign” in Fig 19d, there are very similar values mixed together across the slide with similar shades of grey for both “malignant” and “other or benign”. The similar values show that the patch classifier is unsure in these areas. The second row of Fig 19 shows examples of patches from this slide in decreasing probability of being malignant. Fig 19e shows the patch with the highest probability of being malignant. The probability of Fig 19f is very slightly higher than 0.5 so is classified as malignant whereas the probability of Fig 19g is very slightly lower than 0.5 so this patch would be classified as benign. Finally Fig 19h is a patch that the classifier is very confident is benign.

The slide classifier has incorrectly classified this “malignant” slide as “other or benign”. However, the probabilities predicted by the slide classifier are “other or benign” = 0.456, “malignant” = 0.455, “insufficient” = 0.09. The probabilities are almost identical for “malignant” and “other or benign”, so demonstrating the slide classifier is unsure about this slide. The probabilities predicted by the slide classifier can be used to sort slides and presented to pathologists. This slide would therefore be presented before most of the slides the model is sure are benign and so although wrongly classified would still be higher in a prioritised list.

Given these errors it seems that further work on improving the patch classification stage would lead to improved overall results. The overall patch classification accuracy for the final model, on the validation set is 88.8%, on the test set is 87.6%,showing there is room for improvement of the patch classifier. Perhaps by increasing the patch classification accuracy it would be possible to improve the results both where the classifier is wrong and unsure. This work was carried out in parallel with work on cervical biopsies from the same source, many model settings for the patch classification were first examined in the cervical case and to save time it was assumed the best options would travel across to the endometrial version, for example investigating different CNNs for patch classification. It is possible that these parameters require more optimisation for the endometrial case.

Improving the patch classification accuracy would allow the use of much simpler slide classification algorithms. For example, a perfect patch classifier would mean any slides with malignant patches would be classed as malignant. This is how pathologists view slides, where any malignant tissue means that the slide is malignant. At the current patch classification accuracy such simple slide classifiers based on number of percentage of malignant patches gave poorer results than those presented. A reason for this could be that the model gives a probability of a patch being malignant, to which a threshold is then applied to determine if it is to be classed as “malignant” or not. A patch slightly over the threshold might be classed as “malignant”, whereas in reality the classifier is unsure of the classification. The uncertainty could be because the patch is not similar to something in the training set, or perhaps only a very small part of the patch looks odd. This uncertainty is very naturally dealt with by clinicians but is harder for algorithms to negotiate. It is one of the reasons multiple thresholds are included in the current slide classification algorithms.

In addition we can examine the subcategories of the slides that are incorrectly classified to see if there are indications where the modelling is going wrong. Of the final six incorrectly classified “malignant” slides in the validation set, four are “hyperplasia with atypia” and two are “adenocarcinoma”. “Adenocarcinoma” slides are approximately twice as common in the dataset and so in a random sample of slides you would expect to find more “adenocarcinoma” slides than “hyperplasia with atypia”. The fact that more “hyperplasia with atypia” slides were incorrectly classified may indicate the model is less accurate at classifying “hyperplasia with atypia” than “adenocarcinoma”. In the test set the 9 incorrectly classified malignant slides are 3 “adenocarcinoma”, 3 “hyperplasia with atypia”, 2 “sarcoma” and 1 “other”. As “sarcoma” and “other” slides are rare in the dataset (see Table 1) these are over represented, “hyperplasia with atypia” slides are also slightly over represented and “adenocarcinoma” under represented. These are similar results to the validation set showing that the model is less accurate at “hyperplasia with atypia” than “adenocarcinoma”. This may indicate that the patch classifier needs more examples of “hyperplasia with atypia”, “sarcoma” and “other” patches for training. There are relatively few “sarcoma” and “other” patches available so increased sampling of these may help the patch classifier. There are a large number of “hyperplasia with atypia” patches but this category represents a spectrum and there can be disagreement between pathologists where the boundaries are on the spectrum. Perhaps more examples of “hyperplasia with atypia” patches are required to capture the wider variation in the spectrum and would also help to compensate for imperfect ground truth resulting from the disagreement between pathologists.

### 4.3 Potential slide classification improvements

The slide classification is a combination of feature extraction from the patch classification heatmaps and a simple machine learning model to distinguish between “malignant”, “other or benign” and “insufficient” categories. Alternative slide classification approaches were trialled early in the development process, for example a simple binary classification of “malignant” or “not malignant” and a two stage algorithm first identifying the “malignant” slides and then identifying the “insufficient” slides. The model trained with three categories performed better than these alternative approaches so we concentrated on optimising the three category slide classification models. The slide classification step is necessary to deal with noise and imperfections from the patch classifier. If improvements are made to the patch classifier, it would be worth reconsidering these simpler approaches that more closely mimic the processes of the human pathologists.

In Section 3.3 we also tried to train a CNN on the heatmaps, the CNN was not as accurate as the more hand engineered version, but this was largely due to the “insufficient” class. One of the disadvantages in training a fully supervised model is the need for annotations, which are time intensive for pathologists to produce. The slide classification stage however only needs a slide level label which are much quicker to produce. If more “insufficient” slide images and labels are available, it is therefore possible to create heatmaps from the existing patch classification model without the need for additional annotation and so improve the slide classification model. A much larger number of heatmaps available for training could improve the CNN based slide classification above the current feature extraction based model. It is thought that increasing the number of slides available for training, particularly for the “insufficient” class, which make up only 7% of the current training set, is likely to be the best way to further improve the slide classification results. It should be noted that the largest interobserver confusion was between “other or benign” and “insufficient” and so there is lack of clarity in the ground truth of these classes. Therefore even with a much larger number of slides to train distinguishing between “other or benign” and “insufficient” will still prove to be a more challenging problem.

There is also scope for improvements in the patch classification and preprocessing pipeline. Alongside this fully supervised work we have been examining a weakly supervised approach [28] on the same data using multiple instance learning, the results have not matched the fully supervised work, but are close. Increasing the amount data for training of the fully supervised model requires annotations which are slow to produce and require large amounts of time from pathologists. The benefit of the weakly supervised approach is that it doesn’t require these annotations just the slide label and so if very many more slides images can be acquired it is possible that the weakly supervised approach would match or overtake the fully supervised approach. A disadvantage of weakly supervised methods such as multiple instance learning is they do no directly predict which areas of the tissue are malignant or benign, which is why this paper concentrated on the fully supervised methods.

## 5 Conclusions

The goal was to train a model that could be used to automatically sort histopathology slides of endometrial biopsies into “malignant” or “other or benign” thus allowing prioritisation of the pathology workload and reducing time to diagnosis for patients with cancer. The final model applied a grey scale based tissue detector and a pretrained blood and mucus detector to reduce the amount of image processing required. The slide was then split into 1024 × 1024 pixel patches which were passed into a patch classifier. A heatmap was built up from the predicted probability of the patches, features were extracted from the heatmaps and passed into an XGBoost slide classifier. The final model was able to accurately classify 90% of all slides correctly and 97% of slides in the “malignant” class.

In order to automate diagnosis further improvements in accuracy are required. Three initial steps were identified as most likely to improve this model, improving the preprocessing pipeline, improving the patch classifier and improving the slide classifier. The most immediate opportunity for improving the results is get more examples of “insufficient” slides, this would likely improve the CNN based slide classifier and allow it to overtake the current more engineered version. Several options were identified for improving the preprocessing pipeline for tissue detection, blood or mucus detection and patching, these would have the added benefit that preprocessing pipelines could transfer across different tissue types and also apply to weakly supervised approaches. Producing extra annotations is too time expensive to be plausible and so strategies for improving patch classification would be based on increasing the patches used for training, for example through extensive augmentation or much finer patch striding.

These results can be used to prioritise a queue of slides, allowing pathologists to review the slides identified as being “malignant” and therefore speed up results. In order to speed up pathologists workflow further the next step is to accurately identify the subclasses in both “malignant” and “other or benign” which would for allow automatic generation of reports.
